# MD simulations reveal the basis for dynamic assembly of Hfq–RNA complexes

**DOI:** 10.1016/j.jbc.2021.100656

**Published:** 2021-04-20

**Authors:** Miroslav Krepl, Tom Dendooven, Ben F. Luisi, Jiri Sponer

**Affiliations:** 1 Institute of Biophysics of the Czech Academy of Sciences, Brno, Czech Republic; 2 Department of Biochemistry, University of Cambridge, Cambridge, United Kingdom; 3 MRC-LMB, Cambridge, United Kingdom

**Keywords:** Hfq protein, molecular dynamics, RNA-binding protein, RNA metabolism, protein–nucleic acid interaction, Crc protein, ARN repeats, dynamic recognition, 4BPh, type-4 base–phosphate, ARN, adenine–purine–any nucleotide, Crc, catabolite repression control, PDB, Protein Data Bank, sRNA, small regulatory noncoding RNA molecule, vdW, van der Waals

## Abstract

The conserved protein Hfq is a key factor in the RNA-mediated control of gene expression in most known bacteria. The transient intermediates Hfq forms with RNA support intricate and robust regulatory networks. In *Pseudomonas*, Hfq recognizes repeats of adenine–purine–any nucleotide (ARN) in target mRNAs *via* its distal binding side, and together with the catabolite repression control (Crc) protein, assembles into a translation–repression complex. Earlier experiments yielded static, ensemble-averaged structures of the complex, but details of its interface dynamics and assembly pathway remained elusive. Using explicit solvent atomistic molecular dynamics simulations, we modeled the extensive dynamics of the Hfq–RNA interface and found implications for the assembly of the complex. We predict that *syn*/*anti* flips of the adenine nucleotides in each ARN repeat contribute to a dynamic recognition mechanism between the Hfq distal side and mRNA targets. We identify a previously unknown binding pocket that can accept any nucleotide and propose that it may serve as a ‘status quo’ staging point, providing nonspecific binding affinity, until Crc engages the Hfq–RNA binary complex. The dynamical components of the Hfq–RNA recognition can speed up screening of the pool of the surrounding RNAs, participate in rapid accommodation of the RNA on the protein surface, and facilitate competition among different RNAs. The register of Crc in the ternary assembly could be defined by the recognition of a guanine-specific base–phosphate interaction between the first and last ARN repeats of the bound RNA. This dynamic substrate recognition provides structural rationale for the stepwise assembly of multicomponent ribonucleoprotein complexes nucleated by Hfq–RNA binding.

Hfq is a conserved RNA-binding protein and a pleiotropic regulator of translation and RNA stability in diverse bacteria. Some of its best studied roles are to suppress translation of target mRNAs by annealing them with small regulatory noncoding RNA molecules (sRNAs) (1, 2, 3, 4) or by directly binding an A-rich sequence in the 5′-untranslated region of mRNAs (5, 6). Six Hfq protomers assemble to form a hexameric, ring-like chaperone (Fig. 1*A*). The hexamer can bind RNAs *via* three surfaces (7, 8), commonly termed as proximal, distal, and rim faces or sides (9, 10). Furthermore, the intrinsically disordered C-terminal regions can also interact with RNA and autoregulate the activity of Hfq (11, 12). The homo-oligomeric nature of Hfq favors recognition of nucleotide repeats in target RNAs, such as the ARN-triplet repeat motif (where A is an adenine and R and N are a purine and any nucleotide, respectively), which binds on the distal side (Fig. 1*B*) (13).

**Figure 1:**
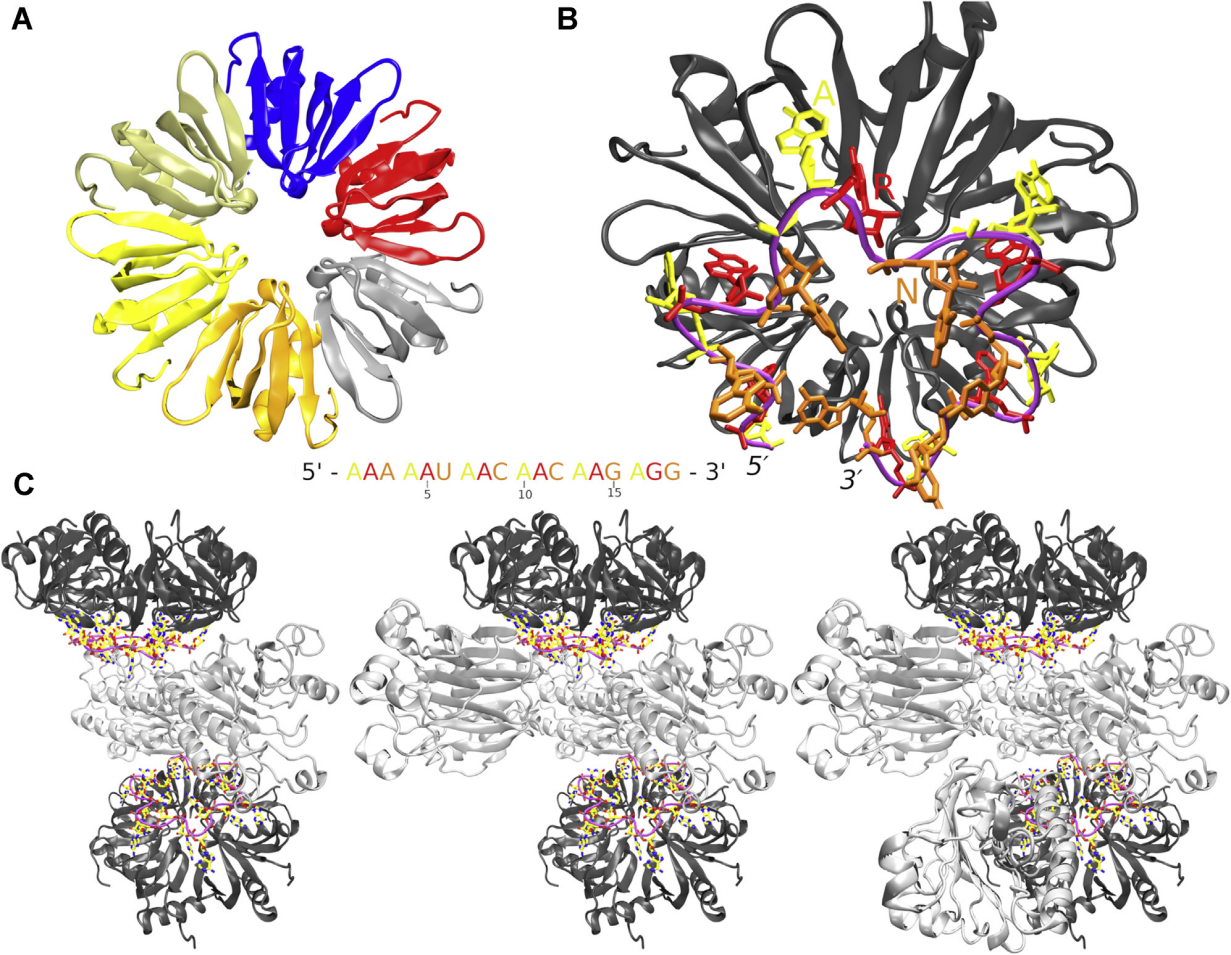
**Hfq architecture and assembly into higher order ribonucleoprotein particles.***A*, the structure of the *Pseudomonas aeruginosa* Hfq hexamer with differently colored monomeric units and its RNA-binding distal side highlighted in *pink*. *B*, a tilted view into the distal side with the *amiE*_*6ARN*_ mRNA engaged. The A, R, and N nucleotides are colored in *yellow*, *red*, and *orange*, respectively. The RNA sequence is specified. The Hfq protein is colored in *dark gray*, and the RNA backbone is in *pink*. *C*, the quaternary complex formed by Hfq, Crc, and *amiE*_*6ARN*_. From *left* to *right*, the quaternary complex can contain two, three, or four Crc units (*light gray*), respectively. Crc, catabolite repression control.

In the gram-negative bacterium *Pseudomonas aeruginosa*, Hfq was identified as a versatile contributor to metabolic regulation (14) whose influences on different pathways are facilitated *via* interactions with other proteins (15). One such partner is the catabolite repression control (Crc) protein (16). In *Pseudomonas*, Crc is responsible for directing the metabolic pathways toward preferring succinate over other potential carbon sources (17). The mechanism of Hfq and Crc cooperation involves binding of both proteins to ARN repeats in the 5′-untranslated region of target mRNAs, thus repressing expression of enzymes involved in alternative metabolic pathways (14). When succinate is depleted, the sRNA CrcZ is expressed (18) and proceeds to sequester Hfq and Crc from their mRNA targets, allowing the mRNAs of alternative metabolic genes, such as the *amiE*, to be translated (6).

Crc has no intrinsic RNA-binding or Hfq-binding capabilities of its own (16), yet it can bind to Hfq–RNA complexes and strengthen Hfq interactions with target RNAs (14). The structural basis of this cooperative action has been unraveled by cryo-EM, which showed Hfq, Crc, and a short segment of *amiE* mRNA forming a quaternary complex (19). The structure of this complex encompasses two Hfq hexamers, each complexed with an RNA octadecamer containing six ARN repeats, the *amiE*_*6ARN*_ fragment. At a minimum, there is a homodimer of two Crc proteins positioned between the two Hfq–RNA complexes. We henceforth refer to this structure as the quaternary complex (Fig. 1*C*). Depending on availability, up to two additional Crc proteins can be recruited into the quaternary complex (19).

The cryo-EM structures of the quaternary complex reveal a recognition motif seen in earlier X-ray structures of Hfq complexed with polyadenine RNAs (8, 20). In the crystal structures, the A and R nucleotides of ARN repeats are specifically bound by the Hfq, whereas the N nucleotides are bulged away from Hfq and interact with the neighboring crystallographic cells. The same RNA recognition pattern is present in the quaternary complex except that the N nucleotides are instead engaged in nonspecific interactions with the Crc proteins (19).

In this study, we used atomistic molecular dynamics (MD) simulations to explore the conformational variation of the Hfq–RNA binary complex and the higher order quaternary complexes formed with Crc. Our MD simulations utilize a set of carefully calibrated molecular mechanics models (21, 22) that have been applied in many studies of protein–RNA complexes with predictive power (21, 23, 24, 25, 26, 27, 28). MD allows the study of atomic movements at spatiotemporal resolutions inaccessible to any currently available experimental method and can help rationalize experimental observations (21). Although simulation timescales are generally short, well-executed simulations can provide insights into biomolecular dynamics that are not apparent from static models obtained by structural experiments, for which the data are typically time- and ensemble-averaged (23, 29).

Biomolecular dynamics can be invaluable for understanding the nature of complex intramolecular interfaces, such as those of protein–RNA complexes, where it underpins binding affinities, specificities, and formation rates. The interface dynamics between biomolecules can involve competing local conformational substates rather than a fixed geometry (30), resulting in *dynamic recognition*. The substates associated with the dynamical ensemble may be important for the detailed mechanisms of the process of binding and unbinding (31). Dynamic recognition can be biologically significant as it could facilitate highly specific recognition of RNAs by a protein and would provide a mechanism by which a large pool of cellular RNAs can be interrogated with speed, specificity, and high affinity for target sequences. Different interaction intermediates can be preferred by different binding partners in quaternary complexes. We propose that Hfq must utilize a form of dynamic recognition because its *in vivo* RNA cycling at both proximal and distal sides was shown to be disproportionally fast relative to its low-nanomolar RNA-binding affinity, as measured by *in vitro* experiments (32, 33).

Our results suggest that extensive equilibrium local dynamics indeed occur at the distal side interface of the Hfq–RNA complex and offer a concrete example of a conformational switch that can influence rates of translocation along a length of RNA. Namely, the first nucleotide in each ARN repeat (*i.e.*, the adenines) can undergo frequent *syn*/*anti* flips, before Crc binding. The *anti* and *syn* conformations are supported by Hfq *via* unique adenine-specific interactions in both positions. The frequency of the flips is lowered upon formation of the quaternary complex, that is, after Crc is bound, and the *syn* conformation becomes less favored. We also identify a previously unknown binding pocket at the distal side of Hfq which can weakly bind the N nucleotides of the ARN repeats in the absence of Crc. Finally, we suggest a potential assembly pathway in which the Crc initially recognizes an intramolecular RNA interaction in the *amiE*_*6ARN*_.

## Results

### Design, stability, and reproducibility of the MD simulations

The simulations of Hfq–RNA and quaternary complexes showed no loss of structural compactness and integrity, which indicates good performance of the force field (21) and sufficient quality of the experimental structures (8, 19) used as the starting states for the simulations. For such large systems, we did not expect to achieve a full thermodynamic convergence within affordable computational time (34). In fact, such convergence is not fully achieved even in longer MD simulations of much smaller systems such as RNA tetraloops and tetranucleotides (35). Nevertheless, even without achieving full quantitative convergence, the MD simulations can provide a wealth of information about the RNA systems (21) inaccessible to experimental methods. The simulations presented here can be considered qualitatively converged in a sense that the same simulation trends were observed in multiple independent parallel trajectories of the individual systems (Table 1). In addition, we extended selected simulations up to 5, 10, or 15 μs (Table 1), observing the same trends even on these longer timescales. The analyses presented in the main text are based mainly on these extended trajectories. For the remaining systems, the analyses were performed on a combined simulation ensemble and are described in Supporting information.

**Table 1:** List of simulations

Simulation name	Source PDB^a^	#Hfq	#RNA	#Crc	Number of simulations × length (μs)
Quaternary complex simulations					
2Hfq_2Crc_2RNA	6o1k	2	2	2	1 × 5, 2 × 1
2Hfq_3Crc_2RNA	6o1l	2	2	3	2 × 1
2Hfq_4Crc_2RNA	6o1m	2	2	4	1 × 5, 1 × 1
2Hfq_4Crc_2RNA_A_A_-*syn*^b^	6o1m	2	2	4	2 × 5, 1 × 1
Hfq_Crc1_RNA^c^	6o1k	1	1	1	2 × 2
Hfq_Crc2_RNA^c^	6o1k	1	1	1	2 × 2
Hfq_2Crc_RNA	6o1k	1	1	2	2 × 1
2Hfq_4Crc_2RNA_noHBfix^d^	6o1m	2	2	4	1 × 1
Quaternary complex simulations–modified systems					
2Hfq_2Crc_2RNA_G18C^e^	6o1k	2	2	2	2 × 1
2Hfq_2Crc_2RNA_circ^e^	6o1k	2	2	2	2 × 1
2Hfq_2Crc_2RNA_ext^e^	6o1k	2	2	2	2 × 1
Other simulations					
Hfq_RNA	6o1k	1	1	0	1 × 15, 2 × 1
Hfq_RNA_allG_R_^f^	6o1k	1	1	0	1 × 10, 2 × 2
Hfq_RNA_I30A^e^	6o1k	1	1	0	2 × 1
Hfq_RNA_pol-A^g^	3gib	1	1	0	1 × 2
2Crc	6o1k	0	0	2	2 × 1
2Crc_4jg3	4jg3	0	0	2	2 × 1
4Crc	6o1m	0	0	4	1 × 2
Hfq_RNA_noHBfix^d^	6o1k	1	1	0	2 × 2
Hfq_RNA_^2×^HBfix^h^	6o1k	1	1	0	1 × 2

Crc, catabolite repression control; PDB, Protein Data Bank.

a PDB ID of the experimental structure which was utilized as the initial structure. In some simulations, only selected parts of the experimental structure were used (see Selection of initial structures and Fig. S1).

b All bases of the “A” nucleotides within the ARN repeats were modified to be in *syn* conformation before the simulation start.

c Crc1 and Crc2 refer to the Crc proteins that bind near and away from G_18_, respectively (Fig. S1).

d Simulations were done without the HBfix (see System building and simulation protocol and Supporting information).

e In “G18C”, “circ”, “ext”, or “I30A” simulations, the G_18_ nucleotide of the RNA was replaced with C_18_, the RNA chain was circularized by covalently connecting the 5′- and 3′-nucleotides *via* a newly modeled phosphate, the RNA was extended from its 3′-end by adding nucleotides U_19_ and G_20_, or I30 was mutated into alanine, respectively.

f All the “R” nucleotides within the ARN repeats were modified to be guanosines.

g A circular polyadenine RNA octadecamer was bound to Hfq.

h HBfix with 2 kcal/mol penalty was utilized (see System building and simulation protocol and Supporting information).

**Figure 2:**
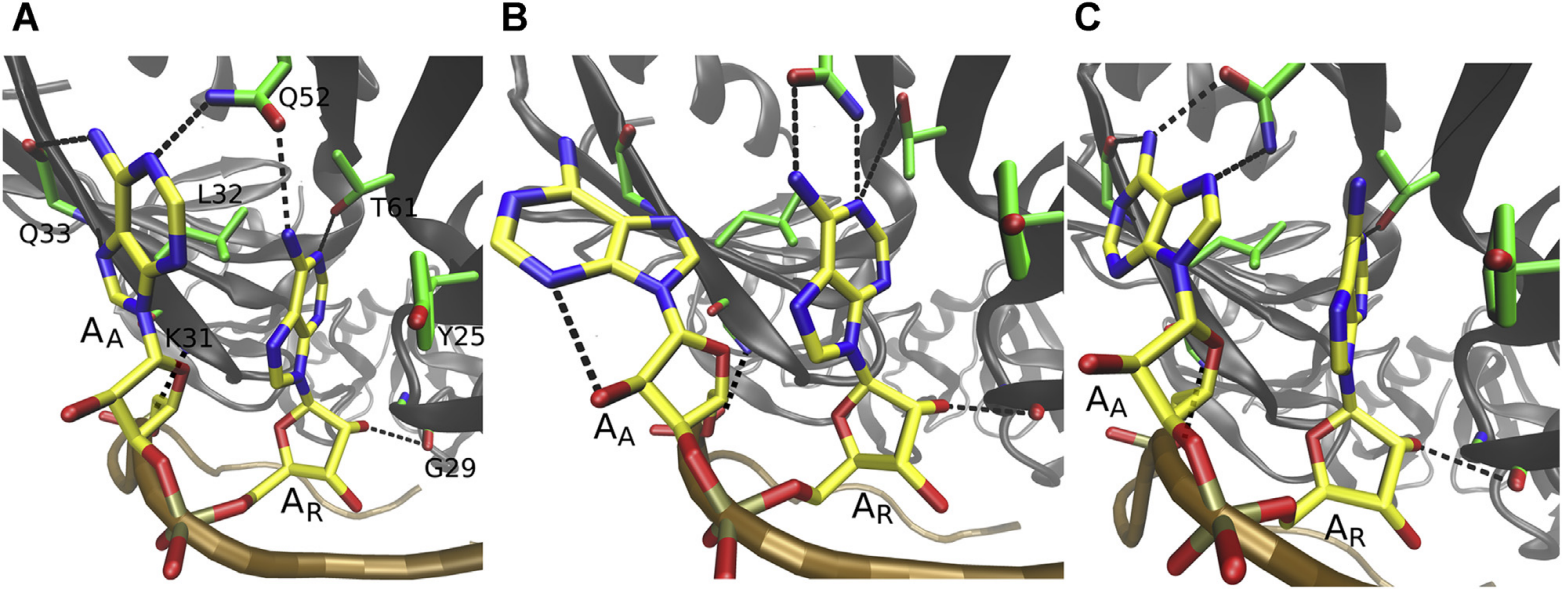
**Reversible *syn*/*anti* flip on the distal face of Hfq.** Interactions observed for the A_A_/A_R_ binding pocket with the A_A_ base in (*A*) *anti*, (*B*) a transition intermediate, and (*C*) *syn*. The interacting nucleotides and amino acids are labeled on the *left panel*. Carbon atoms are colored *yellow* and *green* and the backbones traced in *brown* and *dark gray*, in RNA and Hfq, respectively. The *black dashed lines* indicate H-bonds. Unless specified otherwise, we use this labeling and color coding in rest of the text. See below for description of the A_A_/G_R_ binding pocket.

The bound RNA in the quaternary complex consists of six ARN trinucleotide repeats while the Hfq itself is a hexamer (Fig. 1*B*). All ARN motif nucleotides in the positions “A” (includes A_1_, A_4_, A_7_, A_10_, A_13_, and A_16_) and “R” (includes A_2_, A_5_, A_8_, A_11_, A_14_, and G_17_), respectively, are bound in identically organized binding pockets, and their positions are clearly defined by H-bonds, base stacking, and van der Waals (vdW) interactions (Fig. 2*A*). The pockets exhibited highly similar behavior in simulations, and their protein–RNA interactions were all maintained with only reversible fluctuations (Tables S1 and S2). To simplify descriptions, we will collectively refer to the first and second nucleotides of the repeats as either A_A_ or A_R_/G_R_, with the lower index signifying the nucleotide’s position within the ARN repeat. For example, notation Q33(N)–A_A_(N7) describes backbone amide nitrogen of residues Q33 in the individual Hfq chains forming H-bonds with N7 of RNA nucleotides A_1_, A_4_, A_7_, A_10_, A_13_, or A_16_. Wherever the behavior significantly differed among the repeats, we refer directly to the specific nucleotides by their residue numbering (Fig. 1). To help distinguish Crc amino acids, they are labeled with an accent (*e.g.*, R162′).

### Simulations of isolated Hfq–RNA reveal *syn*/*anti* flips of the A_A_ nucleotides

**Figure 3:**
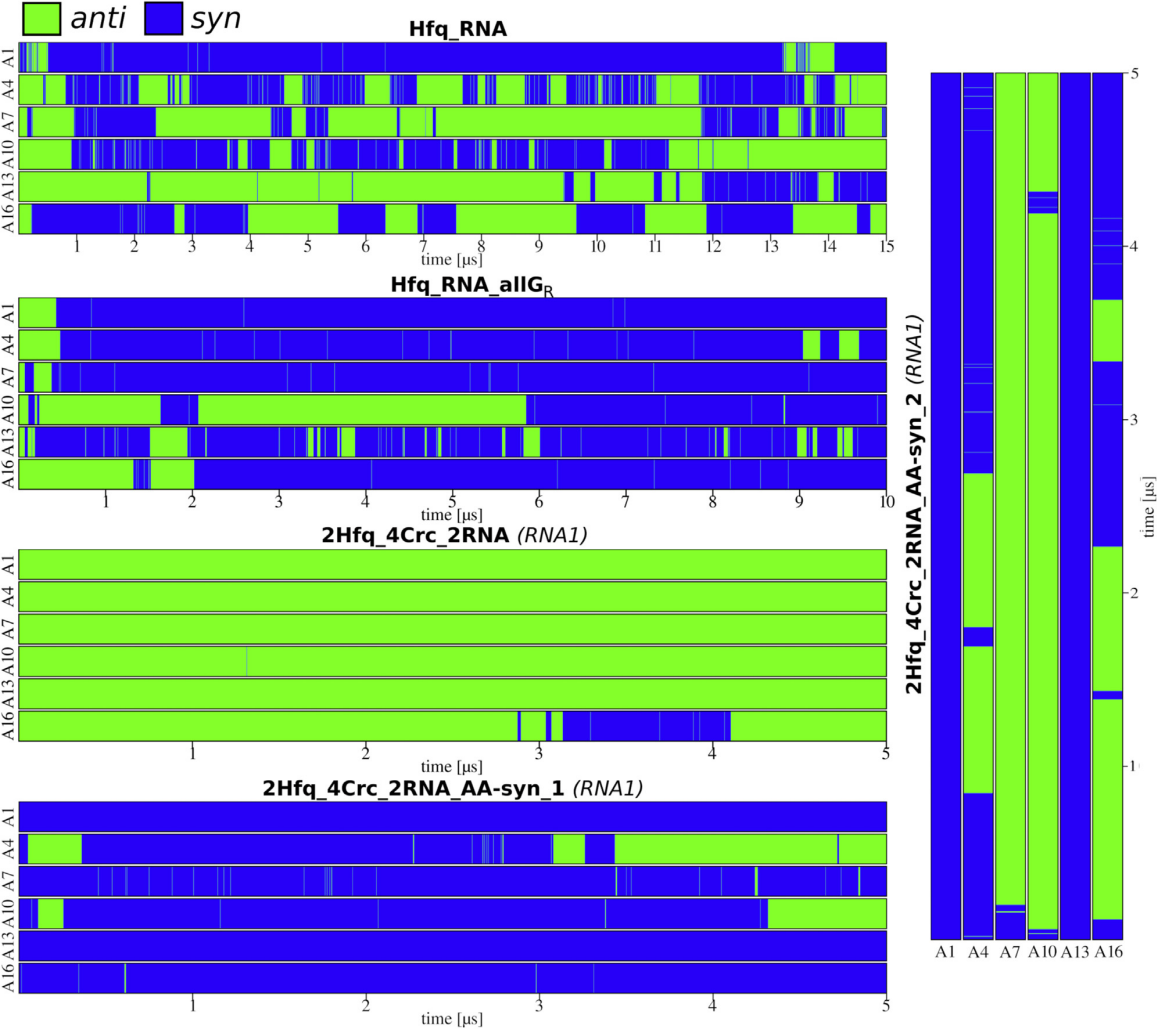
**Time development of the *anti* (*green*)/syn (*blue*) of the A**_**A**_**nucleotides in selected simulations.** For the graphs of the remaining simulations and of the second RNA molecules of systems containing two RNAs, see Fig. S2. See Table 1 for details of the individual systems.

The most significant conformational change observed in the simulations of Hfq–RNA complexes, in absence of Crc, was the dynamic equilibrium between *anti* and *syn* conformations of the A_A_ nucleobases. Most strikingly, the *anti*-A_A_ and *syn*-A_A_ nucleotides established interactions with the same amino acids. Structurally, this was possible by the N7 atom replacing the N1 atom as the H-bond acceptor and vice versa, whereas the second hydrogen of the N6 amino group was utilized as a H-bond donor (Fig. 2). There is also a single water bridge between A_A_ and A_R_, which is formed solely with A_A_ in *anti* (Fig. 3*B*). This water bridge is present in the X-ray structure of poly-A RNA bound to Hfq (8) and was regularly observed in all our MD simulations as long as A_A_ was in *anti* while being abolished in *syn*. Finally, there is a close presumable repulsive atomic contact between O4′ atom of A_A_ ribose and the backbone carbonyl of K31 associated with the *anti* conformation of A_A_ (Fig. 3*B*). In MD simulations, the K31(O)-A_A_(O4′) distance increased when A_A_ flipped into *syn*, thus relieving the repulsion. The average simulation time between the flips differed significantly among the individual simulations and A_A_ nucleotides, ranging from tens to hundreds of nanoseconds (Fig. 3; Fig. S2). The transition intermediate of these flips (Fig. 2*B*) was stabilized by a temporary formation of an intranucleotide H-bond, and the transition time of the flips was in the range of tens of picoseconds. In each simulation, several back-and-forth *syn*/*anti* flips were observed for every A_A_ nucleotide with few exceptions (Table 2; Table S3). We note the relatively large variability of *syn/anti* populations among individual A_A_ nucleotides. After comparing multiple simulation trajectories (Table S3), we conclude that it reflects randomness of simulation sampling and the fact that the *syn/anti* dynamics are not synchronized across the six ARN repeats.

**Table 2:** Transitions between the *anti* and *syn* conformations of individual A_A_ nucleotides in selected MD simulations^a^

Simulation name and length	I: number of transitions/*syn* population^b^ II: time until first transition (ns)^c^ III: Average lifetimes of *syn*/*anti* (ns)^d^						
		A_1_	A_4_	A_7_	A_10_	A_13_	A_16_
Hfq_RNA – *15 μs*	I	14/0.95	39/0.63	15/0.30	22/0.62	7/0.24	11/0.54
	II	17	800	140	905	2210	212
	III	752/25	235/131	240/522	385/214	272/819	1148/871
Hfq_RNA_allG_R_ – *10 μs*	I	1/0.96	5/0.91	3/0.97	5/0.47	27/0.85	3/0.82
	II	426	487	57	120	70	373
	III	–	3036/298	4868/132	938/1062	356/59	4095/905
2Hfq_2Crc_2RNA – *5 μs*	I	0/0.00	2/0.57	0/0.00	0/0.00	0/0.00	4/0.44
		0/0.00	0/0.00	0/0.00	0/0.00	3/0.31	3/0.91
	II	–	1122	–	–	–	38
		–	–	–	–	483	431
	III	–	2844/1077	–	–	–	544/564
		–	–	–	–	777/1148	2284/215
2Hfq_4Crc_2RNA – *5 μs*	I	0/0.00	0/0.00	0/0.00	0/0.00	0/0.00	2/0.19
		0/0.00	0/0.00	0/0.00	0/0.00	0/0.00	2/0.10
	II	–	–	–	–	–	2873
		–	–	–	–	–	174
	III	–	–	–	–	–	342/994
		–	–	–	–	–	292/1031
2Hfq_4Crc_2RNA_A_A_-*syn*_1 – *5 μs*	I	0/1.00	11/0.59	4/0.99	3/0.84	0/1.00	2/0.99
		0/1.00	1/0.99	6/0.56	2/0.98	0/1.00	0/1.00
	II	–	66	3440	111	–	608
		–	4954	785	654	–	–
	III	–	267/262	1243/9	1392/275	–	2497/6
		–	–	463/318	2439/123	–	–
2Hfq_4Crc_2RNA_A_A_-*syn*_2 – *5 μs*	I	0/1.00	4/0.65	1/0.04	3/0.03	0/1.00	6/0.51
		0/1.00	3/0.89	1/0.84	3/0.25	0/1.00	0/1.00
	II	–	858	195	66	–	123
		–	636	4224	84	–	–
	III	–	1091/864	–	95/2405	–	637/817
		–	2218/189	–	423/933	–	–

a The two lines in the “2RNA” simulations each describe one of the two RNA molecules contained in these systems. Crc’s sterically obstruct flips of A_1_ in 2Crc and of A_1_ and A_13_ in 4Crc systems, respectively.

b Number of *syn*/*anti* transitions (in any direction) and the populations of the two states with one and zero corresponding to all-*syn* and all-*anti*, respectively. The A_A_ nucleotide was considered to be in *syn* and *anti* when its χ dihedral angle was −30° to 150° and 150° to 330°, respectively. We disregarded transitions lasting less than 300 ps.

c Simulation time in which the first *syn*/*anti* transition (in either direction) occurred. The “–” symbol indicates that no transition occurred.

d Average simulation time that A_A_ nucleotide remained in *syn* and *anti*, respectively, before flipping. The lifetimes are not stated when there was only a single or no transition observed.

### Crc attenuates *syn/anti* flips of the A_A_ nucleotides

**Figure 4:**
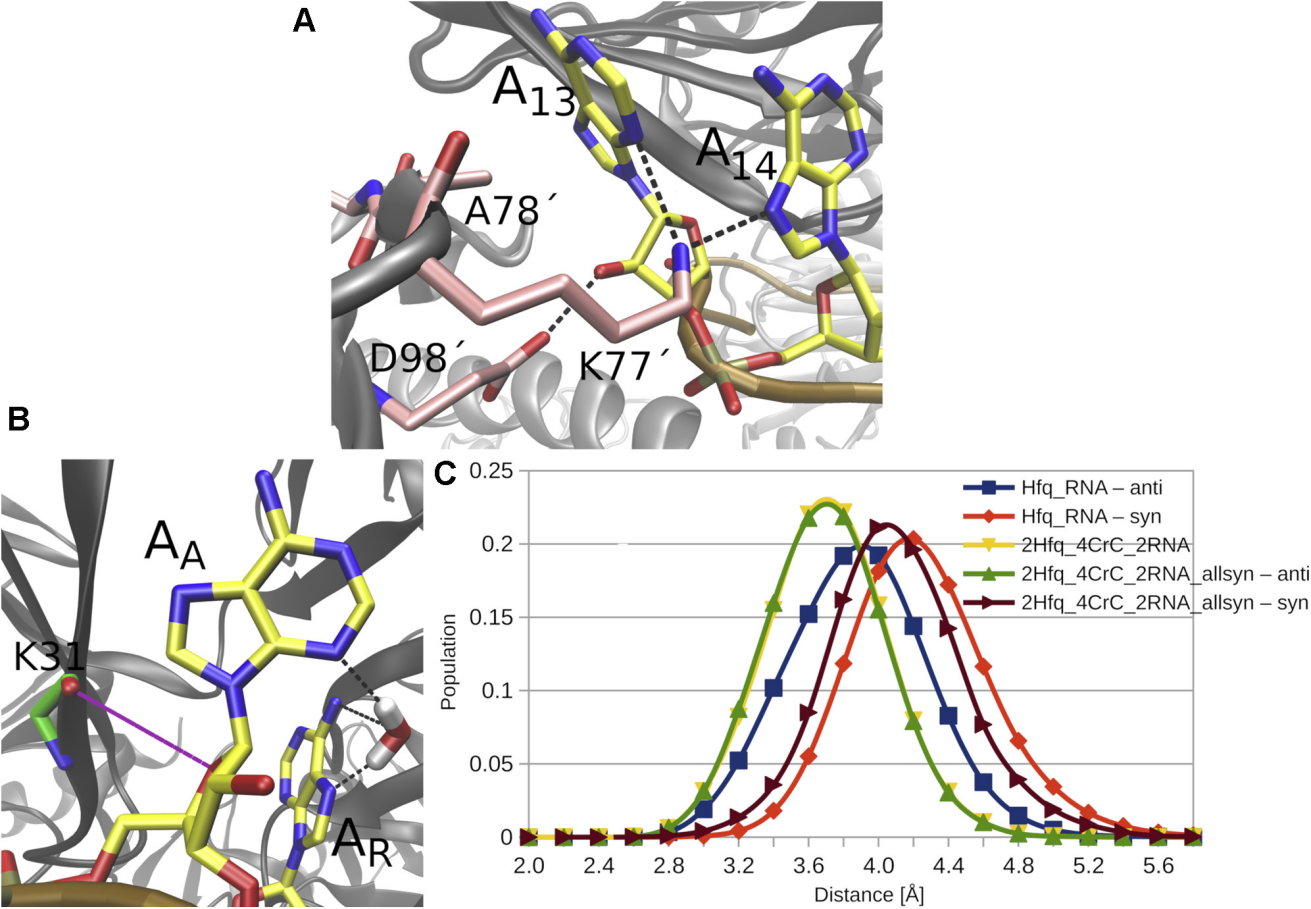
**Recruitment of Crc impedes conformational switching on the Hfq distal face.***A*, binding of Crc proteins sterically obstructs *syn/anti* flips, stabilizes the *anti* conformation of A_13_ nucleobase, and blocks formation of the transition structure in the simulations. The A_1_ nucleobase was similarly immobilized by another Crc protein. *B*, the *anti* conformation of every A_A_ is associated with a water bridge toward A_R_ and a repulsive interaction between the O4′ atom of ribose and the backbone carbonyl of K31. Flip of A_A_ into *syn* relieves the repulsion but disrupts the water bridge. *C*, normalized histograms of the average K31(O)–A_A_(O4′) distances observed in selected simulations for A_A_ nucleotides in either *syn* or *anti* conformations. The presence of Crc produces shorter distances. See Table 1 for details of the individual systems. Crc, catabolite repression control.

The *syn/anti* flips were strongly reduced when Crc is bound to the Hfq–RNA intermediate (Table 2 and Fig. 3) for the unmodified experimental structure (19) where all A_A_s are in *anti*. For nucleotides A_1_ and A_13_, this can be explained by the Crc proteins forming vdW contacts and H-bonds that sterically block the flips and stabilize the *anti* conformation, respectively (Fig. 4*A*) (19). For the rest of the A_A_ positions, Crc forms extensive nonspecific contacts with the phosphate groups immediately downstream (Fig. S3). The *syn* conformation of A_A_ is associated with dihedral angle transitions of this backbone suite (36) but not the *anti* conformation (Fig. S4). This indirectly promotes the *anti* conformation as the non-specific contacts with Crc restrict the available conformational space for such dihedral angle transitions. The overall atomic fluctuations of this backbone suite are also generally lower with Crc (Fig. S5). For both *syn* and *anti* conformations, the K31(O)-A_A_(O4′) distance was shorter in the presence of Crc than in isolated Hfq–RNA (Fig. 4*C*), that is, Crc pushes the A_A_ nucleotide deeper into the A pocket. This could affect the *syn*/*anti* balance as a tighter geometry is associated with the *anti* conformation. Flipping of the A_A_ base into *syn* to relieve the K31(O)-A_A_(O4′) repulsion then becomes less favorable when Crc is bound. On the other hand, the penalty for the loss of the water bridge (Fig. 4*B*) upon flipping into *syn* remains the same in both systems. In conclusion, rather than simply blocking the flips, the Crc could also promote the *anti* conformation by subtly shifting the free-energy balance among multiple interactions within and around the A_A_ binding pocket.

### Prior flipping of A_A_ nucleotides into *syn* alters protein–RNA interface with Crc

The experimental structures used in this study have all the A_A_ nucleotides in the *anti* conformation. In a quaternary complex where we flipped all the A_A_ bases into *syn* before the simulation start (2Hfq_4Crc_2RNA_A_A_-syn simulations; see Table 1), we observed some A_A_ bases returning to *anti* on the simulation timescale (Table 2 and Fig. 3). This strongly contrasts with the simulations where the A_A_ bases were in *anti* from the start (Crc attenuates syn/anti flips of the AA nucleotides) and subsequently showed no signs of flipping into *syn*. However, often the A_A_ bases in the 2Hfq_4Crc_2RNA_A_A_-syn simulations which flipped into *anti* would once again flip into *syn* in time, seemingly contradicting the idea that Crc promotes *anti* or suppresses the flips. For many of the more short-lived *anti* states (Fig. 3), this was because not all of the interactions with Hfq (Fig. 2) had properly formed after A_A_ flipped into *anti*. This never occurred in simulations without Crc and suggests Hfq’s ability to seamlessly accommodate the spontaneous flips (Fig. 2) is limited by Crc. We also observed lower stability and alterations of the Crc–RNA interactions in the 2Hfq_4Crc_2RNA_A_A_-syn simulations (Table S4). Even for repeats where these interactions were not lost, the flips back into syn were also often preceded by temporary disruption of the local Crc–RNA interactions (Fig. S6). This suggests that *a priori* flipping A_A_ into *syn* perturbs the interface with Crc and this disturbance is not fully relaxed in the course of our simulations. This allows subsequent *syn*/*anti* flips in both directions similar to those observed in simulations without Crc. We also suspect there could be some degree of synchronization between the flips of individual A_A_ nucleotides originating from general destabilization of the Crc–RNA interface although this could not be decisively established from our simulations.

### Dynamics of the A_A_/G_R_ binding pocket differs compared with the A_A_/A_R_ consensus

**Figure 5:**
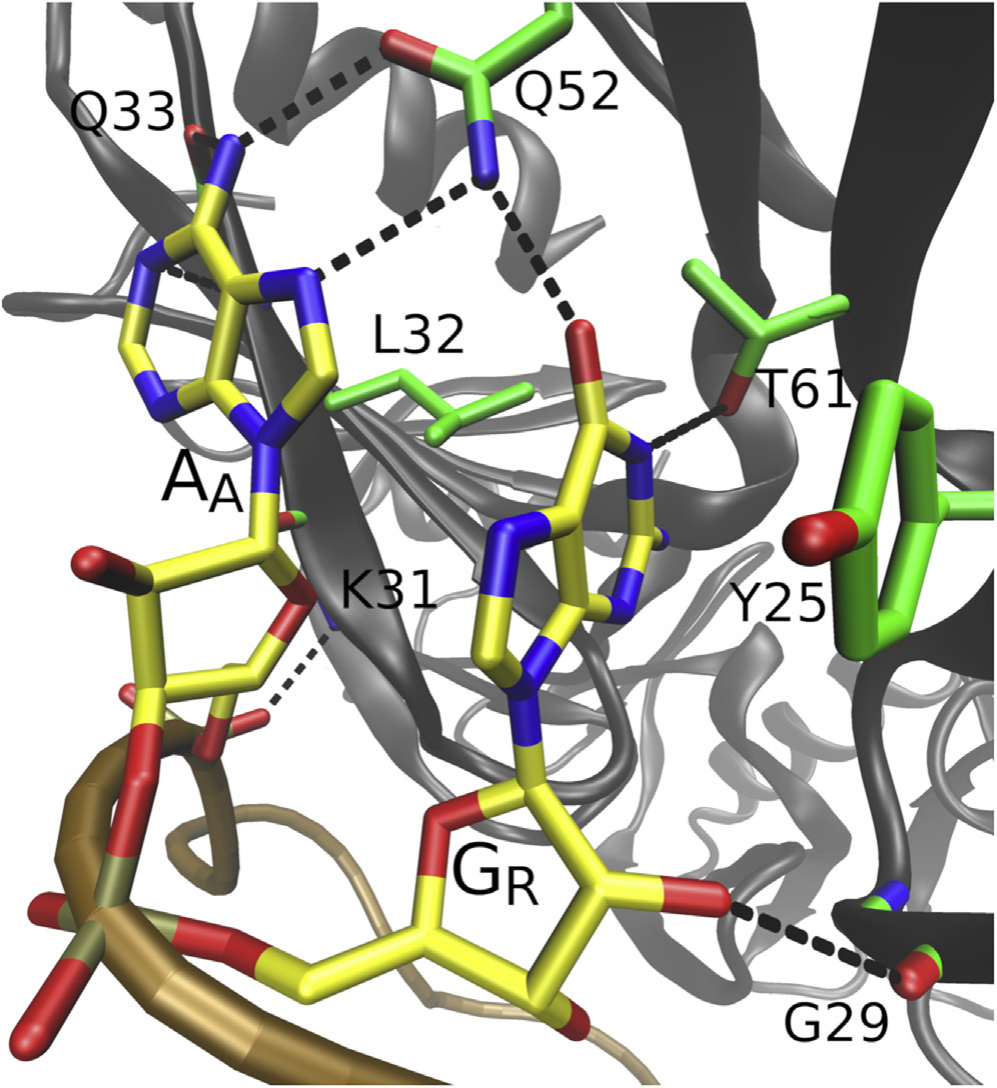
**A**_**A**_**nucleotide in *syn* with G**_**R**_**as the succeeding nucleotide.** Q52 side chain could form simultaneous interaction with both nucleotides in simulations, significantly stabilizing the *syn* conformation of A_A_ when guanosine was the succeeding nucleotide.

The second nucleotide of the ARN repeats can be either adenosine or guanosine, collectively referred to as A_R_ or G_R_. The G_R_ nucleotides form interactions with the same Hfq residues as the A_R_ nucleotides (Fig. 2), both in the experimental structure and in simulations. However, the simulations reveal a striking difference in dynamics of the binding pocket and the associated *syn*/*anti* flips of the preceding A_A_ nucleotide when G_R_ is present instead of the A_R_. Namely, in simulations of the Hfq–RNA complex where we replaced all the A_R_ nucleotides with G_R_ (Table 1), the *syn* population of the A_A_ nucleotides was significantly increased (Table 2 and Fig. 3). We suggest the reason for this is formation of an interaction with the Q52 side chain that can simultaneously interact with A_A_(N6), A_A_(N7), and G_R_(O6) atoms only when A_A_ is in *syn* (Fig. 5).

### The simulations predict a third binding pocket for the N nucleotides on the distal side of Hfq

**Figure 6:**
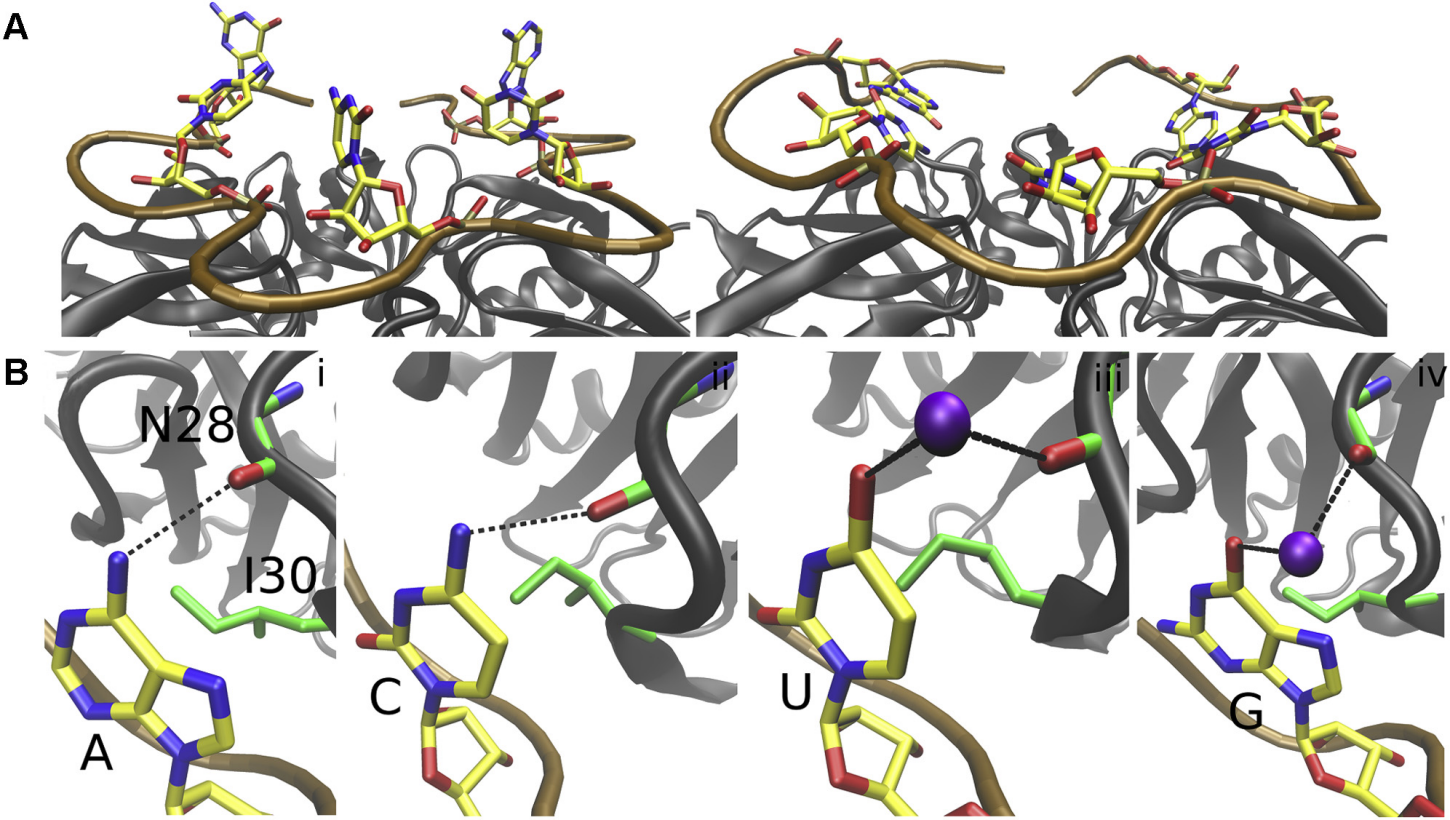
**A distal side pocket for the N position of the ARN motif.***A*, the bases of the N nucleotides of the ARN repeats in the *amiE*_*6ARN*_ mRNA are bulged away from the distal side of Hfq in the quaternary complexes (*left*) (19). In simulations of the isolated Hfq–RNA systems without any Crc, they all bend toward Hfq (*right*). *B*, all the bases (i–iv) formed vdW interaction with I30 and either H-bond or ion-bridge interaction with the backbone of N28. This arrangement is a putative binding pocket for N nucleotides at the distal side of Hfq, the N-pocket. The K^+^ are shown as *lilac spheres*. The *black dashed lines* indicate H-bonds or ion bridges. ARN, adenine–purine–any nucleotide; Crc, catabolite repression control; vdW, van der Waals.

In the cryo-EM structures of the quaternary complex (19), the N nucleotides of all ARN repeats, except G_18_, have their bases turned away from the distal side of the Hfq to interact with Crc. The N nucleotides are positioned similarly in the X-ray structure of isolated Hfq bound to poly-A RNA, where they participate in crystal packing (8). In contrast, all N nucleotides bend toward Hfq in our simulations of isolated Hfq–RNA complexes. The nucleotides formed vdW interactions with I30, as well as H-bonding or ion-bridge interactions with the N28(O) atoms of the individual Hfq subunits (Fig. 6 and Table S5). This consistently occurred in all simulations, either with the *amiE*_*6ARN*_ mRNA or poly-A RNA sequence bound (Table 1) and was universally observed for all N nucleotides except G_18_. The simulations thus predict a third binding pocket at the distal side of the Hfq for nondiscriminatory binding of N nucleotides of the ARN repeats (Fig. 6). We henceforth refer to it as the N pocket, in analogy to the previously described A pockets and R pockets, which bind A and R nucleotides, respectively (8). The existence of N pocket binding would be in agreement with the previous report that Hfq mutants lacking I30 have a reduced affinity for RNA sequences that are bound to the distal side (9). Indeed, in simulations of a system where we replaced I30 in every Hfq chain with alanine, the N-pocket binding was either reduced or abolished (Table S6).

In the cryo-EM structures of quaternary complexes (19), as well as in our simulations, the N nucleotides form nonspecific interactions with the Crc partners. These interactions are described in detail in the Supporting information, except for the 3′-terminal G_18_, which is described in The G18/A3 4BPh base–phosphate interaction is stabilized by Crc. In summary, the novel, putative N-pockets could serve as nondiscriminatory, transient ‘status quo’ binding pocket for the N nucleotides, until a partner molecule, such as Crc, engages the Hfq–RNA intermediate.

### The G_18_–A_3_ 4BPh base–phosphate interaction is stabilized by Crc

**Figure 7:**
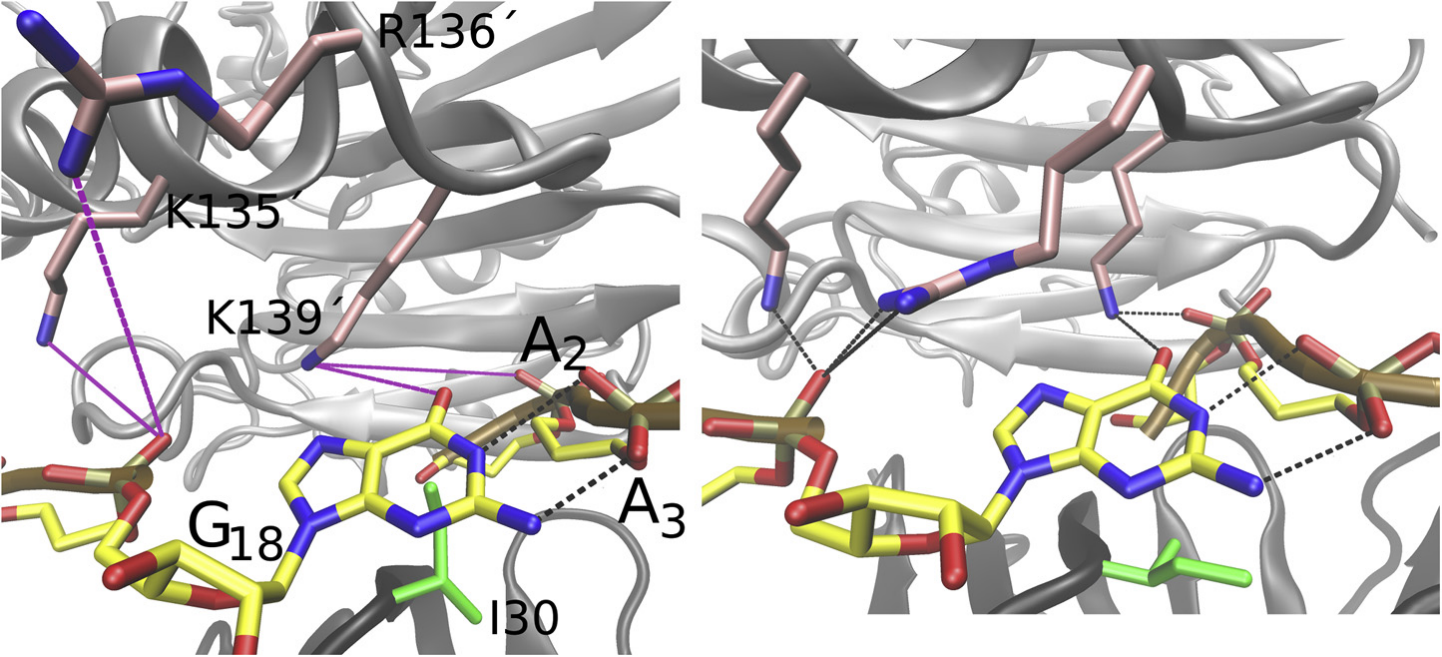
**Registering Crc on the Hfq distal face.** The G_18_ nucleotide as seen in the structure of the quaternary complex (*left*) (19). The *black* and *purple dashed lines* indicate H-bonds and putative H-bonds, respectively. The putative H-bonds between Crc and G_18_ were fully realized in all MD simulations (*right*). The K139′ side chain formed a base-specific interaction with the G_18_ and screened repulsion between the base and the RNA backbone. Crc, catabolite repression control.

The 3′-terminal G_18_ is different from the other N nucleotides in the quaternary complex. First, the G_18_ is not flipped away from the distal side of Hfq in the cryo-EM structure (19). Instead, it forms a vdW interaction with the I30 side chain in a manner similar to the N-pocket binding predicted for the other N nucleotides in isolated Hfq–RNA systems (Fig. 6). Second, the G_18_ is forming a guanine-specific type-4 base–phosphate (4BPh) interaction (37) with the phosphate of A_3_. Third, in the experimental structure, the G_18_ is very close to potentially interact with Crc, namely with the K135′, R138′, and K139′ side chains. These interactions were subsequently formed in MD simulations (Fig. 7 and Table S7). Notably, the K139′ interaction was sequence-specific and seemed to stabilize the intramolecular 4BPh interaction by compensating for the repulsion between the guanine’s O6 atom and the A_2_ phosphate (Fig. 7). This is supported by simulations of the isolated Hfq–RNA system (Table 1), in which the G_18_–A_3_ 4BPh interaction was visibly fluctuating and then lost early in all simulations, along with the G_18_ base vdW interaction toward I30 (Table S7). For more details, see Supporting information.

### *amiE*_*6ARN*_ G_18_ as a putative anchor point for Crc binding to the Hfq–RNA complex

Crc does not dimerize spontaneously (19), which is in agreement with our MD simulations (Supporting information). Yet, a minimum of two Crc proteins is required for full assembly of a quaternary complex with Hfq–RNA (Fig. 1) (19). Therefore, a transiently formed structure with one Crc protein bound to a single Hfq–RNA intermediate could potentially exist at early stages of the quaternary complex formation. We thus simulated two Hfq–RNA–Crc systems that contained either the Crc1 (bound near the G_18_) or the Crc2 protein (Table 1; Fig. S1). We observed reduced fluctuations in relation to the Hfq–RNA for Crc1 compared with Crc2 (Fig. S7). Interestingly, binding of Crc1 alone was sufficient to stabilize the 4BPh base–phosphate interaction and to establish the base-specific interaction with the K139′ formed by the G_18_ nucleobase (Fig. 7 and Table S7).

Next, we explored the dependency of Crc binding on the sequence identity and position of the 3′-terminal nucleotide within the RNA chain. G_18_ is the only N nucleotide recognized specifically by Crc (Fig. 7) and could potentially act as a register-defining marker for its binding. To explore this, we first prepared a system with circularized *amiE*_*6ARN*_ mRNA (Table 1). Building the covalent bond between the two RNA termini necessarily involved disruption of the 4BPh interaction as the G_18_ had to be shifted to make the bond. The G_18_ 4BPh interaction was never reformed in subsequent simulations while no specific interactions between Crc and the G_18_ occurred. The K139′ side chain formed nonspecific interactions with the newly modeled phosphate (Fig. S8). Therefore, the discontinuity in the bound RNA may be essential for proper assembly of the quaternary complex. Next, we prepared systems where we replaced G_18_ with C_18_ (Table 1). There, the C_18_ neither formed base–phosphate interactions nor made any other specific interactions with Crc. It was consequently unstable in its initial position and interacted with the solvent or flipped over to stack with G_15_. Finally, we tested whether the 4BPh interaction could be stable without the G_18_ being a 3′-terminal nucleotide, but still the last nucleotide bound by Hfq, as it would be in a full-length *amiE* mRNA. Thus, we prepared systems where we extended the *amiE*_*6ARN*_ mRNA by two nucleotides, introducing U_19_ and G_20_. The newly modeled nucleotides were positioned in a way to avoid any clashes with the rest of the system. The G_18_ 4BPh interaction remained entirely stable in these simulations.

## Discussion

Hfq is an RNA chaperone involved in numerous regulatory networks in many bacteria. Its diverse functional roles are underpinned by multiple RNA-binding surfaces, each preferring different sequences (9, 10). Here, we examined the structure and dynamics of RNA binding at the distal side of Hfq, which prefers ARN repeats. In 90 μs of MD simulations, we observed significant equilibrium dynamics occurring at the A-pocket of the Hfq–RNA interface, which is attenuated by Crc binding (Fig. 1). In addition, a putative new binding pocket on the Hfq distal side was discovered. Finally, a possible folding pathway of the quaternary complex involving sequence-specific recognition of a 3′-terminal bound guanosine by the Crc is proposed.

### *Syn/anti* flipping in A pockets may contribute to Hfq’s binding strategy

Our simulations revealed *syn/anti* transitions of the A-pocket adenosines (Fig. 2) on a submicrosecond timescale. These transitions were seen for the *amiE*_*6ARN*_ mRNA and for circular poly-A RNA. The adenosines interacted with the same A-pocket amino acids in both *anti* and *syn* conformations (Fig. 2). This strongly suggests that there may be a dynamic equilibrium of *syn* and *anti* A-pocket–bound adenosines existing within the solution structure of the Hfq–RNA complex. There have been previous reports of proteins utilizing *syn/anti* conformational differences to recognize multiple bases (38) or specifically promoting one of the conformations over the other upon binding (39). However, to our knowledge, there are no studies of protein–RNA complexes where a protein could recognize *syn/anti* conformations of a single base equally well *via* a fixed set of amino acids. Importantly, the *syn/anti* flips do not violate the experimentally known specificity of A pockets for adenosines (8) because adenine-specific interactions are formed in both conformations.

The two available X-ray structures of *P. aeruginosa* Hfq (PDB ID: 3gib, 5new) (8, 40) with poly-A RNA bound at its distal side, have the A-pocket–bound adenosines solely in the *anti* conformation. In the structure of *S. aureus* Hfq bound to an RNA A tract *via* its distal side (PDB ID: 3qsu), some of the adenines are in the *syn* conformation, and the study also revealed evidence of *syn*/*anti* flips (41). However, Hfq’s distal sides in *S. aureus* and *P. aeruginosa* are quite different. It should be noted that the assignment of *syn/anti* nucleobase conformers in X-ray structures is often challenging and mistakes can occur even in high-quality structures because of phase errors, resolution limitations, and the time-averaged and ensemble-averaged nature of the data collection (42, 43, 44). The crystal lattice or the cryo temperatures could also enforce the *anti* conformation exclusively (45). Our visual inspection of the electron density maps (8, 40) is consistent with the assigned *anti* conformation, although this does not exclude possibility of phase error.

Populations of the *syn/anti* states predicted by MD simulations may be influenced by potential mild force field imbalances, and thus, the results may not reach quantitative accuracy. However, we suggest that the simulations unambiguously predict that the Hfq A pocket can readily host A in *syn* conformation. The *syn* conformation, even if overpopulated by MD, could represent a transient (higher energy) binding pattern (46) involved in the process of prebinding of RNA to Hfq or in substrate cycling. Such a binding pattern would be undetectable in ground-state experimental structures because of its low population. The *syn* conformation of A-pocket adenosines was significantly more populated when the succeeding R-pocket nucleotide was guanosine instead of adenosine (Fig. 5), suggesting a degree of structural communication between the two pockets. There might also be Hfq cofactors that profit from accessibility of the *syn* orientation or are able to capture the transition conformation (Fig. 2*B*) for binding.

### Crc prefers *anti* conformation of A-pocket nucleotides

Specific protein–RNA interactions between A_A_ nucleobases and Crc directly promoting the *anti* conformation are formed only for the A_1_ and A_13_ (Fig. 4). Hence, at first sight, the Crc should be able to tolerate *syn* in the other A-pocket nucleotides (*i.e.*, A_4_, A_7_, A_10_, and A_16_). Despite this, our simulations of the quaternary complex revealed very few flips toward *syn* for these nucleotides when starting from the experimental structure where they all possess the *anti* conformation (Table 2 and Fig. 3). When we manually flipped all A-pocket adenosines into *syn* before starting the simulations, the A_A_ nucleobases subsequently had the tendency to flip back into *anti* (Table 2 and Fig. 3), despite all the Hfq–RNA *syn* interactions having been established, suggesting that the *syn* conformation is not supported in the presence of Crc. Furthermore, some of the Crc–RNA interactions are destabilized by the *syn* conformation (Table S4). Some flips back into *syn* were observed in case the Hfq interactions did not properly form after flipping into *anti* or during disruptions of some local Crc–RNA interactions (Fig. S6). In other words, the simulation timescale may be insufficient to fully relax the Crc–RNA interface after the initial introduction of the *syn* conformation. We suggest that Crc may promote the *anti* conformation *via* nonspecific interactions it forms with the phosphates downstream of the A_A_ nucleotide (Fig. S3) and by sterically restricting the ability of the A pocket to accommodate the flips that shifts the balance in favor of *anti* (Fig. 4). This observation illustrates the delicate balance of interactions at the Hfq–RNA interface and its potential utilization by various cofactors which may prefer different conformation of A_A_ nucleobases. We acknowledge that the proposed mechanism affecting the *syn*/*anti* balance could include additional components which were not sampled on the timescale of our simulations, such as the partial or complete unbinding of Crc from the quaternary complex.

### The dynamic recognition of RNA could be important for substrate cycling by Hfq

The avid binding affinity of Hfq for various RNA targets appears incompatible with its fast biological binding turnover and cellular response (32, 33, 47). The K_D_s measured *in vitro* are in the subnanomolar range, which would indicate binding half-lives of well over 1 h. In contrast, the cellular responses facilitated by Hfq are on a timescale of 1 to 2 min, suggesting that nascent RNAs are rapidly cycled through the cellular Hfq pool. To reconcile these two observations, it has been suggested that RNA bound by Hfq can be displaced by competitors from the cellular pool in a stepwise process, which was termed as active cycling (32, 33). We note that the extensive dynamics observed in our simulations for RNA bound to the distal side of Hfq could provide an entry point for its displacement by a competing RNA. Importantly, displacement could occur one ARN repeat at a time or even a single nucleotide at a time, as envisaged by the active cycling model (32, 33). Crc and possibly other Hfq-binding proteins could have coevolved to actively suppress the intrinsic dynamics of the Hfq–RNA interface to slow down RNA cycling. However, as our study involved only RNA bound to the distal side of Hfq, we do not claim this interaction fully accounts for the affinity-response discrepancy (32, 33). Moreover, it is likely that we capture only part of the dynamics immediately pertinent to the dominantly bound state because of the 1 to 15 μs timescales of the simulations.

### Hfq provides a weak binding pocket for the N nucleotides

In the quaternary complex (19), the N nucleotides are flipped away from Hfq (Fig. 6) and form many nonspecific interactions with Crc (Supporting information). The exception is G_18_ which forms an intramolecular 4BPh interaction with the A_3_ phosphate (Fig. 7). The N nucleotides are also flipped away in the two X-ray structures of the isolated Hfq–RNA complexes (8, 40) where they form extensive crystal packing interactions.

In our simulations of the isolated Hfq–RNA complexes, all the N nucleotides quickly flipped toward Hfq and formed vdW interactions with I30 side chain and H-bonding or ion bridging with the protein backbone of N28 (Fig. 6). Based on our simulations, we suggest that the I30 and N28 residues might constitute a third binding pocket (N-pocket) at the distal side of Hfq. The N-pocket likely offers weaker contribution to the overall binding affinity than the known A and R pockets (8), given the smaller number of intermolecular interactions that define it. Indeed, the bound nucleotides were very dynamical in our simulations and we regularly observed a drift of the base along the molecular interface formed by vdW interaction with the I30 side chain. Such movement would become especially pronounced during temporary disruptions of the interaction toward the N28. We suggest this type of dynamical binding is realistic as it was reported to be in agreement with NMR data in other protein–RNA systems (48). In addition, we suggest that vdW interactions between nucleobase aromatic faces and other molecules are generally very well described by the utilized AMBER force fields with electrostatic potential–derived charges. This is true even for stacking interactions among nucleobases and with aromatic amino acids. Although these interactions are sometimes described as “π-π” in the literature, rigorous quantum chemical studies (49, 50, 51, 52) showed that AMBER type of force fields provides good description of these interactions. There are no substantial “π-π” orbital effects neglected by the force fields. Rather, these interactions primarily involve electrostatic interaction, London dispersion attraction, and short-range repulsion, all of which can be approximated by the current molecular mechanics model. This does not rule out overstabilization or understabilization of vdW interactions of nucleobases in MD simulations. However, such imbalance would rather involve the solvation and not inaccuracy in description of the direct (intrinsic) vdW contact (21).

The flexible behavior and weak binding explain why the N-pocket RNA recognition is supplanted by crystal packing interactions in the two X-ray structures of the isolated Hfq–RNA complexes (8, 40). However, such disordered-like binding could be useful for dynamic recognition of molecules interacting with the Hfq–RNA surface and could serve as a ‘status quo’ state of the Hfq–RNA intermediate. For example, Crc is recognizing the N nucleotides in a nonspecific manner, whereas other proteins might be able to directly read out their sequence. Owing to their dynamical binding to Hfq, the N-pocket nucleotides are readily available to rapidly establish contacts with other partners. The N-pocket binding observed in our simulations is similar to what is seen in the X-ray structure of *E. coli* Hfq bound to A-rich linker from OxyS sRNA (PDB ID: 4qvc) (53). However, this structure also shows deviations from the ARN repeat consensus and has multiple repulsive and crystal packing interactions affecting its protein–RNA interface. In addition, only three nucleotides could be resolved. Therefore, we opted not to use it for MD simulations.

In summary, we suggest that the “disordered” N-pockets weakly contribute to binding affinity of RNAs to the Hfq in a sequence-independent manner. The dynamical N-nucleotides are at the same time able to quickly establish interactions with other partners, which can already be sequence dependent.

### Crc might sample the ARN repeats during quaternary complex formation, searching for 3′-terminal guanosine

The cryo-EM structure of the quaternary complex (19) shows extensive but base nonspecific interactions between the N nucleotides and Crc, suggesting that the Crc may interact equally well with any of the ARN repeats. It is therefore puzzling how the Crc selects its binding register during the quaternary complex formation or indeed if such selection occurs. The fact that it was possible to resolve a high-resolution structure of the quaternary complex by cryo-EM (19), which relies on averaging of many individual images of the complex, suggests that a specific binding register has been selected.

**Figure 8:**
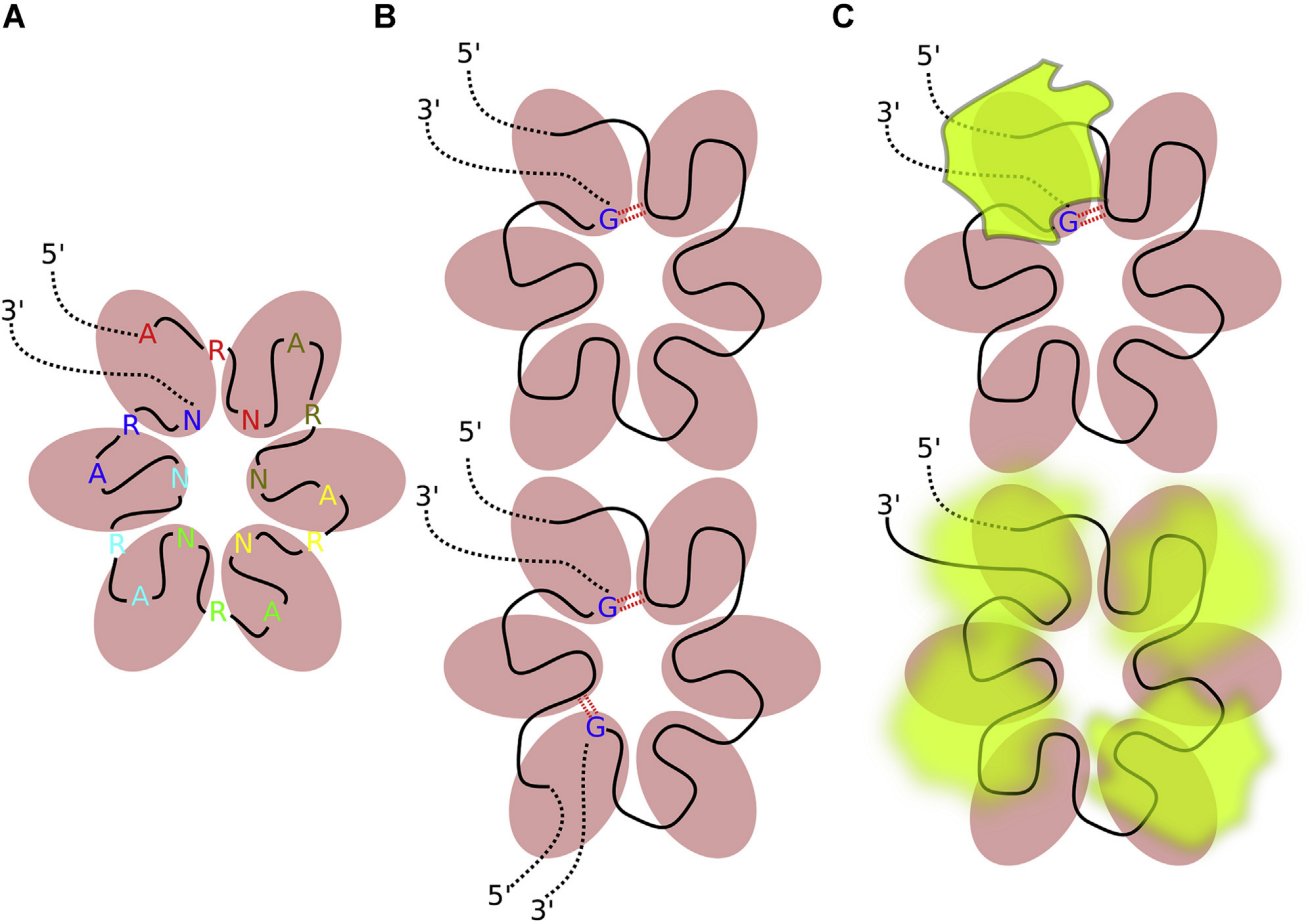
**A model for scanning and locking register in the Hfq–Crc–RNA assembly.***A*, cartoon of the ARN repeat–rich RNA binding to Hfq. The Hfq monomeric units are shown as *pink ovals*. The individual ARN repeats are differently colored. *B*, when the N nucleotide from the last ARN repeat bound by Hfq is guanosine, it can potentially form a base–phosphate interaction (*red dotted double line*) with the first repeat (*top*). This could happen when binding a single RNA sequence with six ARN repeats but also with multiple RNA sequences bound at the same time (*bottom*). *C*, the base–phosphate interaction formed by the guanosine could serve as mark for initial Crc binding (*top*). Without this mark, a degenerate multiple-register binding would be the likely result (*bottom*). ARN, adenine–purine–any nucleotide; Crc, catabolite repression control.

Our simulations indicated that the terminal G_18_ nucleotide and its 4BPh interaction toward the A_3_ phosphate might be the marker which, at least in case of the *amiE*_*6ARN*_ mRNA, provided a specific binding site for Crc. The guanine-specific 4BPh interaction is the strongest base–phosphate interaction occurring in folded RNAs (37). The simulations additionally predict a guanine-specific protein–RNA interaction between G_18_ and the K139′ side chain (Fig. 7). To establish both of these interactions, the guanosine does not need to be the 3′-terminal nucleotide, but merely the last nucleotide bound by the Hfq after which the RNA chain exits the Hfq’s distal side. This would be the case in, for example, a full-length *amiE* mRNA, or during binding of multiple shorter RNA segments rich in ARN repeats, such as the CrcZ (Fig. 8) (14). In either case, recognition of these 3′-terminal guanine-specific interactions by Crc would be enough to overcome binding register degeneracy in the quaternary complex, as the binding sites of the next Crc would then be predefined by the first. It is however unclear whether preventing the degenerate binding of Crc could have effects on Hfq/RNA/Crc recognition *in vivo* or if it is just a system-specific coincidence which may have helped the resolution of the cryo-EM structures (19). We do not expect that the free-energy gain associated with the 3′-terminal guanosine recognition is large enough to completely abolish other Crc-binding patterns. However, even small increases in affinity could play a role in balancing the overall complex kinetic and thermodynamic networks of interactions in which Hfq is involved. In addition, it is possible that binding of some other proteins could be weakened by the same marker.

## Concluding remarks

In this study, we explore the dynamics of RNA recognition by a conserved and pleotropic RNA chaperone, Hfq. We used state-of-the art atomistic MD simulations to obtain high-resolution insight into local dynamics and substates associated with RNA binding to the Hfq distal site. We show that Hfq partly utilizes dynamic recognition of RNA substrates, a type of molecular recognition that is difficult to fully resolve in structural experiments. However, we suggest that dynamic recognition is likely an important contribution to structural mechanism by which Hfq engages RNA targets with adequate specificity while maintaining high RNA turnover rates in the cell. Upon presentation of a target RNA to effector molecules, such as Crc, this turnover is slowed down significantly, allowing for a downstream cellular response.

## Experimental procedures

### Selection of initial structures

We have used cryo-EM structures of Hfq–Crc–RNA quaternary complexes with molecular composition ratios of 2:2:2, 2:3:2, and 2:4:2 (Protein Data Bank [PDB] IDs: 6o1k, 6o1l, and 6o1m) (19) as starting structures for MD simulations. Models of isolated Hfq–RNA, partially assembled quaternary complexes, and the Crc dimer and tetramer were prepared by removal of subunits from the experimental 2:2:2 structure. The simulations of the Crc dimer were also started based on X-ray structure of the isolated Crc protein (PDB ID: 4jg3) (16) with its dimer structure obtained *via* crystallographic symmetry. We have also used the X-ray structure of *Escherichia coli* Hfq bound to RNA poly-A octadecamer for simulations of the isolated Hfq–RNA system (8). Starting structures for simulations with modified, extended, or circularized RNA sequences or modified nucleobase conformations were prepared by molecular modeling of the experimental structures. A complete list of simulated systems is presented in Table 1 and visualized in Fig. S1.

### System building and simulation protocol

The starting files for MD simulations were prepared in tLeap module of AMBER 18 (54). We have used bsc0χ_OL3_ (*i.e.*, OL3) (55) and ff12SB (56) force fields for description of RNA and protein, respectively; the preference for ff12SB rather than ff14SB in protein–RNA simulations is explained elsewhere (57).

A mild 1 kcal/mol stabilizing HBfix potential (58) was applied to the native H-bond interactions K31(N)–A_A_(OP2) and G29(O)–A_R_(O2′). Both of these H-bonds are present in all available experimental structures of *P. aeruginosa* Hfq bound to RNA-containing ARN repeats (8, 19, 40). The HBfix was applied to reduce likelihood of potential random spurious departures of the trajectories from the experimental geometry in longer simulations due to some force-field imperfectness. It allows us to more efficiently examine, within the affordable simulation time, the dynamics corresponding to the Hfq–RNA bound state as indicated by the experiments. We emphasize that unlike the restrained or targeted explicit solvent MD (or even Gō potentials in coarse-grained modeling (59)), HBfix introduces force potential between two atoms only within the narrow region corresponding to the H-bonding distance. Therefore, although somewhat bolstering these H-bonds by increasing their lifetime, it still allows the system to explore other geometries and should not bias the results derived in our study. Nevertheless, to verify this, we performed control simulations without any HBfix and simulations where we used increased 2 kcal/mol potential. These simulations confirmed that the results presented below do not depend on the use of the HBfix. Further details of the HBfix and of the control simulations are extensively described in Supporting information, together with explanation why the HBfix does not bias the results of the study.

In all simulations, the biomolecular systems were surrounded in a truncated octahedral box of SPC/E water molecules (60) with a minimal distance of 13 Å from the box border. The systems were neutralized and a salt concentration of 0.15 M was established by addition of K^+^ and Cl^−^ atoms (61).

The systems built in tLeap were minimized and equilibrated according to the protocol extensively described in Ref. (62) utilizing the pmemd.MPI module (54). Afterward, the production simulations were carried out with the pmemd.cuda module (63). The typical simulation timescale was 1 μs with selected simulations further extended afterward. We have used the SHAKE protocol along with HMR (hydrogen mass repartitioning) to allow a 4-fs integration step (64, 65). Particle mesh Ewald (66) and periodic boundary conditions were used to handle long-range electrostatics and to prevent the box-border bias. The cut-off distance for Lennard–Jones interactions was set to 9 Å. The Langevin thermostat and Monte Carlo barostat (54) were used to keep the systems at temperature and pressure of 300 K and 1 bar, respectively.

### Analyses

The cpptraj (67) was used to perform analyses of all simulation trajectories, and visual molecular dynamics (68) was used for their visual inspection. Raster3D (69) and POV-Ray were used for preparation of figures. LibreOffice and Inkscape were used to prepare graphs and schemes, respectively. The presence of H-bonds was evaluated based on the donor–acceptor distance and donor–hydrogen–acceptor angle, with 3.5 Å and 120° cutoffs, respectively. For selected simulations, principal component analysis (67) was used to evaluate RNA backbone dynamics and global interdomain movements between the Hfq and Crc. Every fifth frame of the trajectories was used to calculate the coordinate covariance matrix which was then diagonalized and used to obtain the first ten eigenvectors (principal components). The principal components of motion were visualized by projecting them along the utilized simulation frames.

## Data availability

The authors declare that the data supporting the findings of this study are available within the article and its Supporting information. The raw MD simulation trajectories can be obtained from the corresponding author (Miroslav Krepl) upon reasonable request.

## Supporting information

This article contains supporting information (6, 21, 35, 36, 57, 58, 70, 71, 72, 73, 74, 75, 76, 77) (Table S8).

## Conflict of interest

The authors declare that they have no conflicts of interest with the contents of this article.

## References

[1] Vogel J., Luisi B.F. (2011). Hfq and its constellation of RNA. Nat. Rev. Microbiol..

[2] Hoekzema M., Romilly C., Holmqvist E., Wagner E.G.H. (2019). Hfq-dependent mRNA unfolding promotes sRNA-based inhibition of translation. EMBO J..

[3] Azam M.S., Vanderpool C.K. (2017). Translational regulation by bacterial small RNAs via an unusual Hfq-dependent mechanism. Nucleic Acids Res..

[4] Kwiatkowska J., Wroblewska Z., Johnson K.A., Olejniczak M. (2018). The binding of class II sRNA MgrR to two different sites on matchmaker protein Hfq enables efficient competition for Hfq and annealing to regulated mRNAs. RNA.

[5] Vecerek B., Moll I., BLASI U. (2005). Translational autocontrol of the Escherichia coli Hfq RNA chaperone gene. RNA.

[6] Sonnleitner E., Wulf A., Campagne S., Pei X.-Y., Wolfinger M.T., Forlani G., Prindl K., Abdou L., Resch A., Allain F.H.-T., Luisi B.F., Urlaub H., Bläsi U. (2018). Interplay between the catabolite repression control protein Crc, Hfq and RNA in Hfq-dependent translational regulation in Pseudomonas aeruginosa. Nucleic Acids Res..

[7] Schumacher M.A., Pearson R.F., Møller T., Valentin-Hansen P., Brennan R.G. (2002). Structures of the pleiotropic translational regulator Hfq and an Hfq–RNA complex: A bacterial Sm-like protein. EMBO J..

[8] Link T.M., Valentin-Hansen P., Brennan R.G. (2009). Structure of Escherichia coli Hfq bound to polyriboadenylate RNA. Proc. Natl. Acad. Sci. U. S. A..

[9] Mikulecky P.J., Kaw M.K., Brescia C.C., Takach J.C., Sledjeski D.D., Feig A.L. (2004). Escherichia coli Hfq has distinct interaction surfaces for DsrA, rpoS and poly(A) RNAs. Nat. Struct. Mol. Biol..

[10] Brennan R.G., Link T.M. (2007). Hfq structure, function and ligand binding. Curr. Opin. Microbiol..

[11] Santiago-Frangos A., Kavita K., Schu D.J., Gottesman S., Woodson S.A. (2016). C-terminal domain of the RNA chaperone Hfq drives sRNA competition and release of target RNA. Proc. Natl. Acad. Sci. U. S. A..

[12] Wen B., Wang W., Zhang J., Gong Q., Shi Y., Wu J., Zhang Z. (2017). Structural and dynamic properties of the C-terminal region of the Escherichia coli RNA chaperone Hfq: Integrative experimental and computational studies. Phys. Chem. Chem. Phys..

[13] Robinson K.E., Orans J., Kovach A.R., Link T.M., Brennan R.G. (2014). Mapping Hfq-RNA interaction surfaces using tryptophan fluorescence quenching. Nucleic Acids Res..

[14] Sonnleitner E., Bläsi U. (2014). Regulation of Hfq by the RNA CrcZ in Pseudomonas aeruginosa carbon catabolite repression. PLoS Genet..

[15] Van den Bossche A., Ceyssens P.-J., De Smet J., Hendrix H., Bellon H., Leimer N., Wagemans J., Delattre A.-S., Cenens W., Aertsen A., Landuyt B., Minakhin L., Severinov K., Noben J.-P., Lavigne R. (2014). Systematic identification of hypothetical bacteriophage proteins targeting key protein complexes of Pseudomonas aeruginosa. J. Proteome Res..

[16] Milojevic T., Grishkovskaya I., Sonnleitner E., Djinovic-Carugo K., Bläsi U. (2013). The Pseudomonas aeruginosa catabolite repression control protein Crc is devoid of RNA binding activity. PLoS One.

[17] Wolff J.A., MacGregor C.H., Eisenberg R.C., Phibbs P.V. (1991). Isolation and characterization of catabolite repression control mutants of Pseudomonas aeruginosa PAO. J. Bacteriol..

[18] Sonnleitner E., Abdou L., Haas D. (2009). Small RNA as global regulator of carbon catabolite repression in Pseudomonas aeruginosa. Proc. Natl. Acad. Sci. U. S. A..

[19] Pei X.Y., Dendooven T., Sonnleitner E., Chen S., Bläsi U., Luisi B.F. (2019). Architectural principles for Hfq/Crc-mediated regulation of gene expression. Elife.

[20] Nikulin A., Stolboushkina E., Perederina A., Vassilieva I., Blaesi U., Moll I., Kachalova G., Yokoyama S., Vassylyev D., Garber M., Nikonov S. (2005). Structure of Pseudomonas aeruginosa Hfq protein. Acta Crystallogr. D Biol. Crystallogr..

[21] Šponer J., Bussi G., Krepl M., Banáš P., Bottaro S., Cunha R.A., Gil-Ley A., Pinamonti G., Poblete S., Jurečka P., Walter N.G., Otyepka M. (2018). RNA structural dynamics as captured by molecular simulations: A comprehensive overview. Chem. Rev..

[22] Nerenberg P.S., Head-Gordon T. (2018). New developments in force fields for biomolecular simulations. Curr. Opin. Struct. Biol..

[23] Campagne S., Krepl M., Sponer J., Allain F.H.T., Wand A.J. (2019). Chapter fourteen - combining NMR spectroscopy and molecular dynamic simulations to solve and analyze the structure of protein–RNA complexes. Methods Enzymol.

[24] Borišek J., Saltalamacchia A., Gallì A., Palermo G., Molteni E., Malcovati L., Magistrato A. (2019). Disclosing the impact of carcinogenic SF3b mutations on pre-mRNA recognition via all-atom simulations. Biomolecules.

[25] Sharma M., Sharma S., Alawada A. (2019). Understanding the binding specificities of mRNA targets by the mammalian quaking protein. Nucleic Acids Res..

[26] Casalino L., Palermo G., Spinello A., Rothlisberger U., Magistrato A. (2018). All-atom simulations disentangle the functional dynamics underlying gene maturation in the intron lariat spliceosome. Proc. Natl. Acad. Sci. U. S. A..

[27] Palermo G., Casalino L., Magistrato A., Andrew McCammon J. (2019). Understanding the mechanistic basis of non-coding RNA through molecular dynamics simulations. J. Struct. Biol..

[28] Sharma M., Anirudh C.R. (2017). Mechanism of mRNA-STAR domain interaction: Molecular dynamics simulations of mammalian quaking STAR protein. Sci. Rep..

[29] Górecka K.M., Krepl M., Szlachcic A., Poznański J., Šponer J., Nowotny M. (2019). RuvC uses dynamic probing of the holliday junction to achieve sequence specificity and efficient resolution. Nat. Commun..

[30] Ripin N., Boudet J., Duszczyk M.M., Hinniger A., Faller M., Krepl M., Gadi A., Schneider R.J., Šponer J., Meisner-Kober N.C., Allain F.H.-T. (2019). Molecular basis for AU-rich element recognition and dimerization by the HuR C-terminal RRM. Proc. Natl. Acad. Sci. U. S. A..

[31] Borkar A.N., Bardaro M.F., Camilloni C., Aprile F.A., Varani G., Vendruscolo M. (2016). Structure of a low-population binding intermediate in protein-RNA recognition. Proc. Natl. Acad. Sci. U. S. A..

[32] Fender A., Elf J., Hampel K., Zimmermann B., Wagner E.G.H. (2010). RNAs actively cycle on the Sm-like protein Hfq. Genes Dev..

[33] Wagner E.G.H. (2013). Cycling of RNAs on Hfq. RNA Biol..

[34] Krepl M., Havrila M., Stadlbauer P., Banas P., Otyepka M., Pasulka J., Stefl R., Sponer J. (2015). Can we execute stable microsecond-scale atomistic simulations of protein-RNA complexes?. J. Chem. Theor. Comput..

[35] Bergonzo C., Henriksen N.M., Roe D.R., Cheatham T.E. (2015). Highly sampled tetranucleotide and tetraloop motifs enable evaluation of common RNA force fields. RNA.

[36] Richardson J.S., Schneider B., Murray L.W., Kapral G.J., Immormino R.M., Headd J.J., Richardson D.C., Ham D., Hershkovits E., Williams L.D., Keating K.S., Pyle A.M., Micallef D., Westbrook J., Berman H.M. (2008). RNA backbone: Consensus all-angle conformers and modular string nomenclature (an RNA Ontology Consortium contribution). RNA.

[37] Zirbel C.L., Sponer J.E., Sponer J., Stombaugh J., Leontis N.B. (2009). Classification and energetics of the base-phosphate interactions in RNA. Nucleic Acids Res..

[38] Daubner G.M., Cléry A., Jayne S., Stevenin J., Allain F.H.-T. (2012). A syn–anti conformational difference allows SRSF2 to recognize guanines and cytosines equally well. EMBO J..

[39] Kligun E., Mandel-Gutfreund Y. (2015). The role of RNA conformation in RNA-protein recognition. RNA Biol..

[40] Schulz E.C., Seiler M., Zuliani C., Voigt F., Rybin V., Pogenberg V., Mücke N., Wilmanns M., Gibson T.J., Barabas O. (2017). Intermolecular base stacking mediates RNA-RNA interaction in a crystal structure of the RNA chaperone Hfq. Sci. Rep..

[41] Horstmann N., Orans J., Valentin-Hansen P., Shelburne S.A., Brennan R.G. (2012). Structural mechanism of Staphylococcus aureus Hfq binding to an RNA A-tract. Nucleic Acids Res..

[42] Wlodawer A., Minor W., Dauter Z., Jaskolski M. (2008). Protein crystallography for non-crystallographers, or how to get the best (but not more) from published macromolecular structures. FEBS J..

[43] Chou F.-C., Sripakdeevong P., Dibrov S.M., Hermann T., Das R. (2012). Correcting pervasive errors in RNA crystallography through enumerative structure prediction. Nat. Methods.

[44] Krepl M., Blatter M., Cléry A., Damberger F.F., Allain F.H.T., Sponer J. (2017). Structural study of the fox-1 RRM protein hydration reveals a role for key water molecules in RRM-RNA recognition. Nucleic Acids Res..

[45] Atakisi H., Moreau D.W., Thorne R.E. (2018). Effects of protein-crystal hydration and temperature on side-chain conformational heterogeneity in monoclinic lysozyme crystals. Acta Crystallogr. D Struct. Biol..

[46] Ganser L.R., Kelly M.L., Herschlag D., Al-Hashimi H.M. (2019). The roles of structural dynamics in the cellular functions of RNAs. Nat. Rev. Mol. Cell Biol..

[47] Santiago-Frangos A., Woodson S.A. (2018). Hfq chaperone brings speed dating to bacterial sRNA. Wiley Interdiscip. Rev. RNA.

[48] Krepl M., Cléry A., Blatter M., Allain F.H.T., Sponer J. (2016). Synergy between NMR measurements and MD simulations of protein/RNA complexes: Application to the RRMs, the most common RNA recognition motifs. Nucleic Acids Res..

[49] Šponer J., Leszczyński J., Hobza P. (1996). Nature of nucleic acid−base stacking: Nonempirical *ab Initio* and empirical potential characterization of 10 stacked base dimers. Comparison of stacked and H-bonded base pairs. J. Phys. Chem..

[50] Šponer J., Jurečka P., Marchan I., Luque F.J., Orozco M., Hobza P. (2006). Nature of base stacking: Reference quantum-chemical stacking energies in ten unique B-DNA base-pair steps. Chemistry.

[51] Šponer J., Riley K.E., Hobza P. (2008). Nature and magnitude of aromatic stacking of nucleic acid bases. Phys. Chem. Chem. Phys..

[52] Sponer J., Sponer J.E., Mladek A., Jurecka P., Banas P., Otyepka M. (2013). Nature and magnitude of aromatic base stacking in DNA and RNA: Quantum chemistry, molecular mechanics, and experiment. Biopolymers.

[53] Wang L., Wang W., Li F., Zhang J., Wu J., Gong Q., Shi Y. (2015). Structural insights into the recognition of the internal A-rich linker from OxyS sRNA by Escherichia coli Hfq. Nucleic Acids Res..

[54] Case IYB-SDA, Brozell S.R., Cerutti D.S., Cheatham T.E., Cruzeiro V.W.D., Darden T.A., Duke R.E., Ghoreishi D., Gilson M.K., Gohlke H., Goetz A.W., Greene D., Harris R., Homeyer N., Izadi S. (2018). AMBER 18.

[55] Zgarbova M., Otyepka M., Sponer J., Mladek A., Banas P., Cheatham T.E., Jurecka P. (2011). Refinement of the Cornell et al. nucleic acids force field based on reference quantum chemical calculations of glycosidic torsion profiles. J. Chem. Theor. Comput..

[56] Maier J.A., Martinez C., Kasavajhala K., Wickstrom L., Hauser K., Simmerling C. (2015). ff14SB: Improving the accuracy of protein side chain and backbone parameters from ff99SB. J. Chem. Theor. Comput..

[57] Šponer J., Krepl M., Banáš P., Kührová P., Zgarbová M., Jurečka P., Havrila M., Otyepka M. (2017). How to understand atomistic molecular dynamics simulations of RNA and protein–RNA complexes?. Wiley Interdiscip. Rev. RNA.

[58] Kuhrova P., Best R., Bottaro S., Bussi G., Sponer J., Otyepka M., Banas P. (2016). Computer folding of RNA tetraloops: Identification of key force field deficiencies. J. Chem. Theor. Comput..

[59] Noel J.K., Levi M., Raghunathan M., Lammert H., Hayes R.L., Onuchic J.N., Whitford P.C. (2016). SMOG 2: A versatile software package for generating structure-based models. PLoS Comput. Biol..

[60] Berendsen H.J.C., Grigera J.R., Straatsma T.P. (1987). The missing term in effective pair potentials. J. Phys. Chem..

[61] Joung I.S., Cheatham T.E. (2008). Determination of alkali and halide monovalent ion parameters for use in explicitly solvated biomolecular simulations. J. Phys. Chem. B.

[62] Krepl M., Vögele J., Kruse H., Duchardt-Ferner E., Wöhnert J., Sponer J. (2018). An intricate balance of hydrogen bonding, ion atmosphere and dynamics facilitates a seamless uracil to cytosine substitution in the U-turn of the neomycin-sensing riboswitch. Nucleic Acids Res..

[63] Le Grand S., Götz A.W., Walker R.C. (2013). SPFP: Speed without compromise—a mixed precision model for GPU accelerated molecular dynamics simulations. Comput. Phys. Commun..

[64] Ryckaert J.P., Ciccotti G., Berendsen H.J.C. (1977). Numerical-integration of cartesian equations of motion of a system with constraints - molecular-dynamics of N-alkanes. J. Comput. Phys..

[65] Hopkins C.W., Le Grand S., Walker R.C., Roitberg A.E. (2015). Long-time-step molecular dynamics through hydrogen mass repartitioning. J. Chem. Theor. Comput..

[66] Darden T., York D., Pedersen L. (1993). Particle mesh Ewald - an N.Log(N) method for Ewald sums in large systems. J. Chem. Phys..

[67] Roe D.R., Cheatham T.E. (2013). PTRAJ and CPPTRAJ: Software for processing and analysis of molecular dynamics trajectory data. J. Chem. Theor. Comput..

[68] Humphrey W., Dalke A., Schulten K. (1996). VMD: Visual molecular dynamics. J. Mol. Graph..

[69] Merritt E.A., Bacon D.J., Carter C.W., Sweet R.M. (1997). Raster3D: Photorealistic molecular graphics. Macromolecular Crystallography, Pt B.

[70] Zhao J., Kennedy S.D., Berger K.D., Turner D.H. (2020). Nuclear magnetic resonance of single-stranded RNAs and DNAs of CAAU and UCAAUC as benchmarks for molecular dynamics simulations. J. Chem. Theor. Comput..

[71] Mlýnský V., Kührová P., Kühr T., Otyepka M., Bussi G., Banáš P., Šponer J. (2020). Fine-tuning of the AMBER RNA force field with a new term adjusting interactions of terminal nucleotides. J. Chem. Theor. Comput..

[72] Kuhrova P., Mlynsky V., Zgarbova M., Krepl M., Bussi G., Best R.B., Otyepka M., Sponer J., Banas P. (2019). Improving the performance of the RNA amber force field by tuning the hydrogen-bonding interactions. J. Chem. Theor. Comput..

[73] Bottaro S., Bussi G., Kennedy S.D., Turner D.H., Lindorff-Larsen K. (2018). Conformational ensembles of RNA oligonucleotides from integrating NMR and molecular simulations. Sci. Adv..

[74] Szabla R., Havrila M., Kruse H., Šponer J. (2016). Comparative assessment of different RNA tetranucleotides from the DFT-D3 and force field perspective. J. Phys. Chem. B.

[75] Bergonzo C., Henriksen N.M., Roe D.R., Swails J.M., Roitberg A.E., Cheatham T.E. (2014). Multidimensional replica exchange molecular dynamics yields a converged ensemble of an RNA tetranucleotide. J. Chem. Theor. Comput..

[76] Condon D.E., Kennedy S.D., Mort B.C., Kierzek R., Yildirim I., Turner D.H. (2015). Stacking in RNA: NMR of four tetramers benchmark molecular dynamics. J. Chem. Theor. Comput..

[77] Banáš P., Mládek A., Otyepka M., Zgarbová M., Jurečka P., Svozil D., Lankaš F., Šponer J. (2012). Can we accurately describe the structure of adenine tracts in B-DNA? Reference quantum-chemical computations reveal overstabilization of stacking by molecular mechanics. J. Chem. Theor. Comput..

