# Supplementary material for: MD simulations reveal the basis for dynamic assembly of Hfq–RNA complexes
Source: J Biol Chem. 2021 Apr 20;296:100656. doi: 10.1016/j.jbc.2021.100656 (PMC8121710; doi:10.1016/j.jbc.2021.100656)
Supplement: Supplemental Figures S1–S8 and Tables S1–S8 [file mmc1.docx]

# Supporting Information

**Use of HBfix in the MD simulations to moderately bolster sampling of the native state and the justification of such approach.**

MD simulations of protein-RNA complexes starting from known experimental geometries may sometimes deviate from the native state to some non-native conformations, due to accumulation of force-field inaccuracies along the trajectories. This can lead to sampling of spurious long-living substates or even gradual deterioration of the interfaces. The simulations can provide valid information before the problems emerge but probability of such developments increases with the prolongation of the simulation times. Therefore, it is justified to moderately bolster the native state to increase its structural stability, i.e., its lifetime. Such structure-based interventions into simulation trajectories should, however, be made in a way that do not affect the studied properties of the system. They also should not prevent the system to visit any alternative conformations and should be as mild as possible. Therefore, standard restraints and similar approaches are not recommended. In contrast, the recently introduced local HBfix potentials appear suitable for the purpose as they allow mild stabilization of selected H-bonds. In this work, in majority of simulations we introduced a 1 kcal/mol HBfix potential between the hydrogen and acceptor atoms of the K31(N)/A_A_(OP2) and G29(O)/A_R_(O2′) H-bonds (main text Figure 2). The use of HBfix reduced the likelihood of excessive departures from the experimental geometry which could otherwise occur on the extended simulation timescale when multiple interactions within the individual ARN repeats could become disrupted at once.

Structure-specific HBfix is an approach based on locally acting potentials designed to gently modulate stability of native H-bonds in MD simulations ([1](#_ENREF_1),[2](#_ENREF_2)). Each HBfix function is formally composed of two flat-well distance restraints. Their combination produces zero force potential except for the narrow distance range corresponding to a hydrogen-acceptor atom distance of a typical H-bond (2 to 3 Å in this work). In this narrow region, the HBfix produces additional force, in effect increasing stability of the selected hydrogen bond; such potential could also substitute for the missing polarization term. It produces zero force for the alternative conformational states in which the selected H-bond is not present ([1-3](#_ENREF_1)). In other words, the HBfix **does not prevent sampling of alternative states**, although it moderately supports a very few native H-bonds. It increases the lifetimes (and thus populations) of the affected H-bonds but it neither prevents their breakage nor enforces them.

It is important to emphasize that the HBfix is much less intrusive than other commonly used methods such as the restraints, targeted MD, and Gō type potentials, which continuously force simulations into sampling the targeted geometries ([2](#_ENREF_2)). The HBfix approach should not be compared with such methods. The amount of energy used to stabilize the H-bonds is similar to those produced by Gō potentials. However, the HBfix as utilized in this work is applied only to selected interactions whereas all native contacts are stabilized in Gō model calculations. Importantly, the Gō potentials are used in the context of coarse grained modelling where the effect of structure-based bias is much stronger compared to the atomistic explicit-solvent molecular dynamics. We also point out that a potential formally identical to the HBfix has been recently proposed as a method for tuning of the H-bonding in the general RNA force field ([4](#_ENREF_4)).

We wish to point out that the temporary loss of individual protein/RNA interactions (which is attenuated but not prevented by HBfix) could be a realistic development on the simulation timescale. However, simultaneous loss of multiple local interactions in simulations can also expose the RNA to known force-field deficiencies associated with unbalanced description of single-stranded RNA states, such as excessive RNA intramolecular interactions ([4](#_ENREF_4)). Our past experience is that occurrence of these problems typically prevents reformation of the protein/RNA interactions on the simulation timescale, as the RNA establishes excessive self-interactions instead. An ideal solution to the problem would be tuning RNA force field for better description of the single-stranded RNA molecules. Such a solution had been a subject of ongoing and dedicated theoretical research for many years, with numerous studies typically focusing on RNA tetranucleotides as model systems ([4-11](#_ENREF_4)). It still remains a largely unresolved issue as the proposed solutions to improve simulation behavior of single-stranded RNAs typically worsen description of RNAs in other structural contexts. Resolving this issue is outside the scope of this work, which explores structure and equilibrium dynamics of RNA fully bound to just one specific protein. Therefore, we suggest that the use of the HBfix in our case is a justified step to avoid known deficiencies of the force field. The HBfix merely bolsters sampling of the native protein-RNA interface (by reducing likelihood of some random spurious conformational development) but does not introduce significant bias into the studied properties.

It should be noted that, when starting from the experimental structure, bolstering few native interactions at the protein-RNA interface is a more viable approach than trying to, e.g., eliminate the RNA self-interactions. Curation of intramolecular RNA interactions would require a much larger intervention into the force field targeting many more parameters. We probably would need to tune numerous RNA self-interactions (which, in addition, are not know *a priori*) in an iterative manner, including van der Waals RNA self-interactions, which generally is difficult. In this context it is easier to provide moderate bolstering to very few known native H-bonds at the protein-RNA interface. This is the reason why we suggest that the HBfix approach, although not a panacea, is currently the best method to stabilize protein/RNA interfaces in simulations when a tendency to the interface degradation or sampling of long-living spurious substates is suspected.

**Control simulations of the selected Hfq/RNA systems without HBfix.** To demonstrate that the simulation results presented in the main text were not affected by the use of HBfix, we performed control simulations of the most important systems without the use of any HBfix (see above and main text Table 1). These control simulations were shorter in comparison to the HBfix simulations and the simultaneous loss of the protein/RNA interactions did not yet progress towards the force-field deficiencies described above. Importantly, the control simulations revealed the same simulation trends as the simulations with HBfix. Namely, the *syn*/*anti* flips of the A_A_ nucleotides (main text Figure 2) occurred in Hfq/RNA simulations even when HBfix was not applied. It likewise occurred when 2 kcal/mol instead of 1 kcal/mol HBfix was applied to the K31(N)/A_A_(OP2) and G29(O)/A_R_(O2′) H-bonds (Table S3 and Figure S2). The frequency of the *syn*/*anti* flips and their lifetimes varied to the same degree as in the HBfix simulations. The flips were also still suppressed in the 2Hfq_4Crc_2RNA simulations when HBfix was not used. For one of the two RNAs in the system, the exception was the A16 nucleotide, which however, would sometimes flip even in the HBfix simulations. The simulations of isolated Hfq/RNA without HBfix likewise revealed the formation of the binding pocket for the N-nucleotides (main text Figure 6). In conclusion, based on our control simulations without HBfix we conclude that the results presented in the main text were not influenced by the use of the HBfix, as it was supposed to be.

**Crc utilizes the N nucleotides for its binding to Hfq/RNA.** Bases of the N nucleotides except G_18_, as seen in the quaternary complexes, are flipped away from Hfq and interacting with Crc. Namely, in structures containing two, three or four Crc proteins, the A_3_ base is sandwiched between R140´ and R196´ side-chains of Crc. Furthermore, the U_6_ base is forming U_6_(O2)/M156´(N) H-bond. In systems with three or four Crc proteins, the C_9_ is additionally H-bonding with R138´, the C_12_ is forming C_12_(O2)/R140´(N) interaction, and G_15_ stacks with R140´ side-chain. Lastly, there are interactions between Crc and the RNA backbone phosphates of U_6_, A_8_, C_9_, and A_10_ and in systems with three or four Crc proteins also with A_11_, A_13_, and A_14_ (Figure S3). In our MD simulations of the quaternary complexes, we observed a very diverse behavior where some native interactions of the N nucleotide bases were almost entirely maintained while others were less often populated (Table S4). For example, the C_9_ regularly fluctuated between the native H-bond interaction and a stacking interaction with the R138´ side-chain. Another typical simulation behavior was that the base fluctuated between its native interaction with Crc and interaction with the N-pocket of Hfq as seen in simulations of isolated Hfq/RNA (see the main text Figure 6). Interestingly, different behavior of the N nucleotides was observed also between the two RNA molecules within the quaternary complexes (Table S4). Since both protein/RNA interfaces are essentially identical in the starting structures, this indicates a non-converged sampling of the N nucleotides.

**The G_18_/A_3_ 4BPh interaction is unstable without Crc**. The loss of the G_18_/A_3_ 4BPh interaction in isolated Hfq/RNA systems was consistently observed in all attempted simulations whereas the interaction was only once lost in any simulation where Crc was present and bound at this location. Namely, we observed the loss of this interaction in the 5-μs-long simulation of 2Hfq/4Crc/2RNA system, in only one of the two bound RNAs, after ca 1.2 μs of simulation. This is associated with local deformation of the binding pocket and might also be a result of force-field imbalance. We suspect that the reason why the G_18_/A_3_ 4BPh interaction was not subsequently restored in this simulation arises from overstabilization of base stacking interactions in the AMBER force fields ([12](#_ENREF_12)). Likewise, in the simulations of the isolated Hfq/RNA, the G_18_ mostly flipped over to stack with the nearby G_15_ base after losing the 4BPh interaction. This might be further compounded by force field possibly underestimating the stability of the 4BPh interaction as the swift loss of the G_18_ 4BPh interaction in the isolated Hfq/RNA systems, although structurally plausible, is to our opinion perhaps excessively quick. Nevertheless, the stabilization of the 4BPh interaction by Crc is unequivocal and the interaction appears to be specifically recognized by Crc.

**The Crc dimer is unstable per se.** Previous experiments indicated that the Crc does not dimerize in solution ([13](#_ENREF_13)). In agreement with this, we observed a progressive loss of the Crc dimer interface when the dimer is removed from the quaternary complex. The dimer would either be lost completely in simulations or form a variety of non-native inter-protein interactions instead. This process occurred both in simulations started with the Crc dimer or tetramer excised from the Cryo-EM structures of the quaternary complexes as well as in simulations started with the X-ray structure of the Crc with the dimer structure obtained by crystallographic symmetry (see the main text Methods and Table 1). No such behavior was observed for the Crc proteins in simulations where they were embedded within the quaternary complex. The speed of the degradation of native interactions indicates a very fast k_off_ process which suggests that in absence of the other partners the Crc molecules do not readily dimerize or at least do so without preference for a specific dimerization interface.

# Supporting Figures


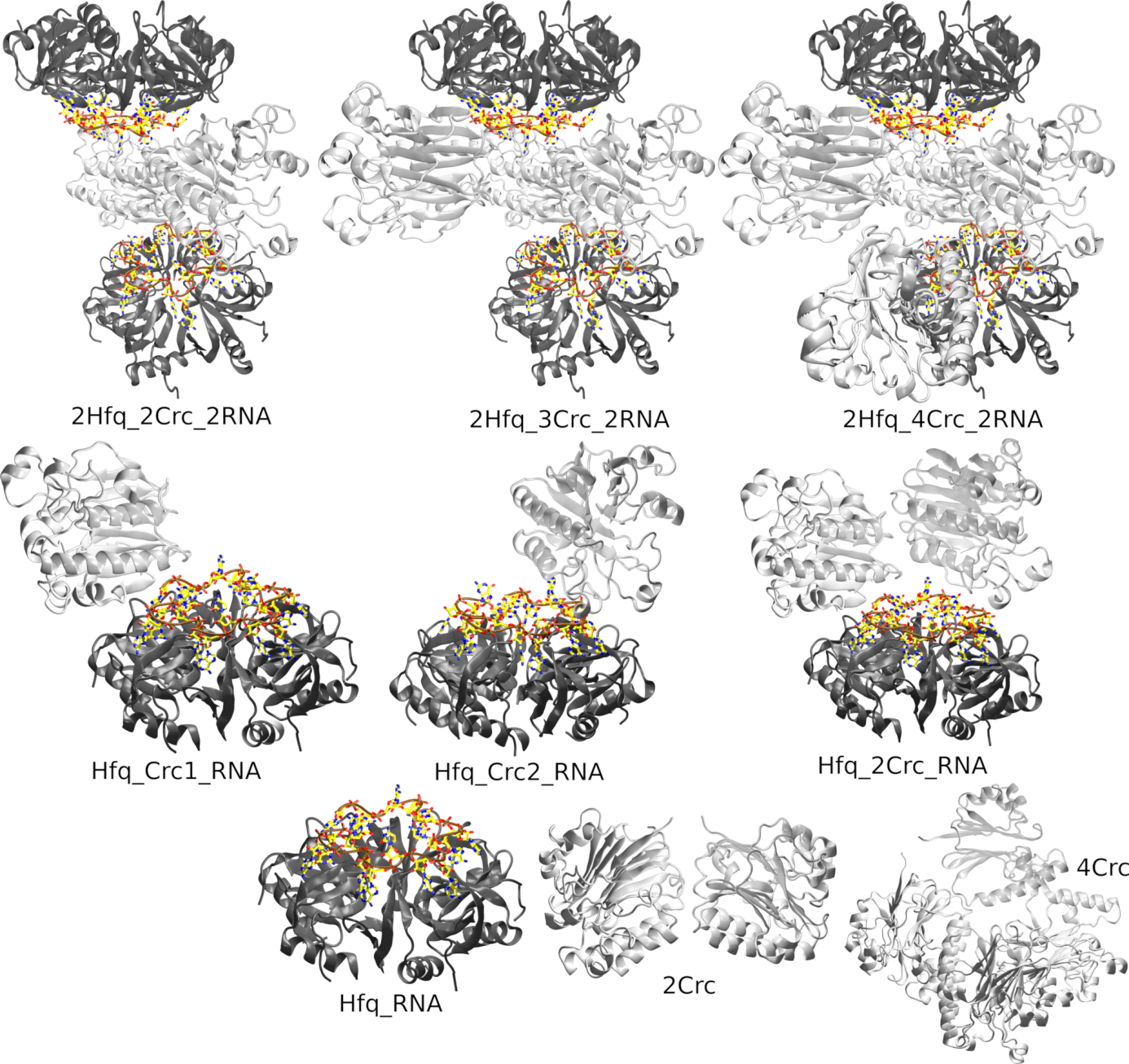


Figure S1. Overview of the structures and molecular compositions of the simulated systems. Hfq and Crc proteins are shown as dark and light gray ribbons, respectively, and RNA is shown in stick representation with its backbone traced in brown.


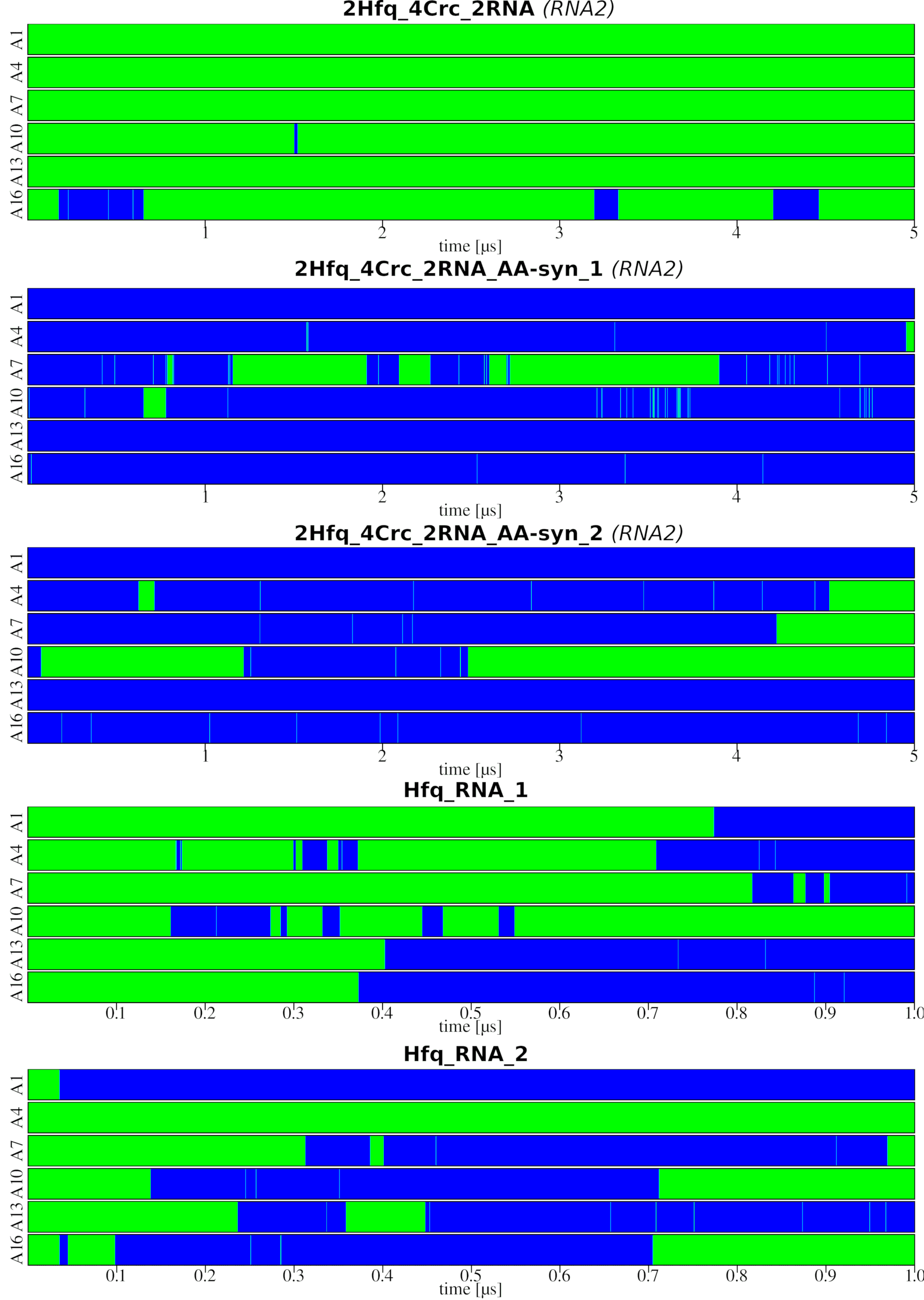


Figure S2. Time development of the *anti* (green) and *syn* (blue) conformations of the A_A_ nucleotides in specified MD simulations. The Figure continues on the following pages.


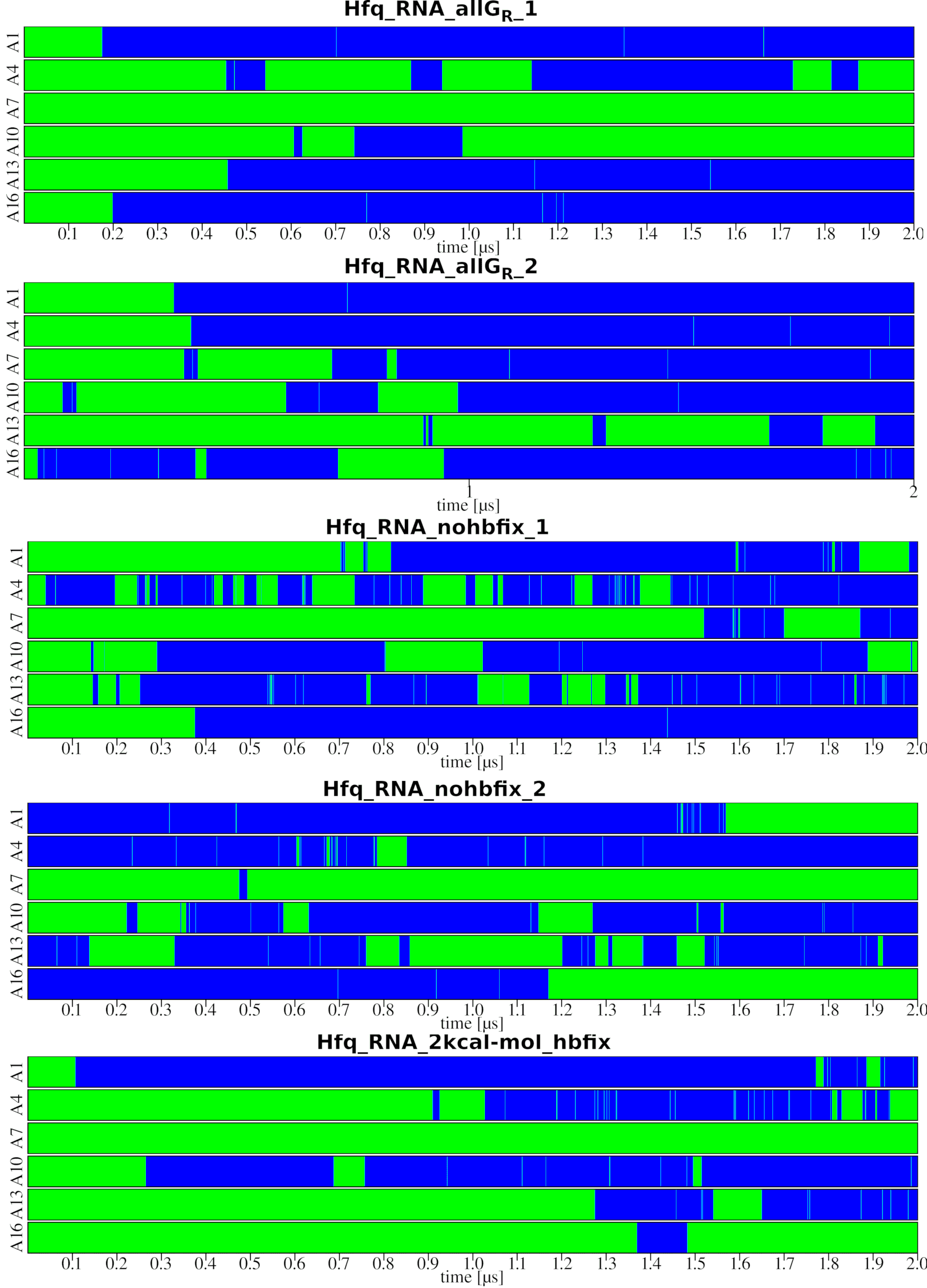


Figure S2. (Continuation from previous page)


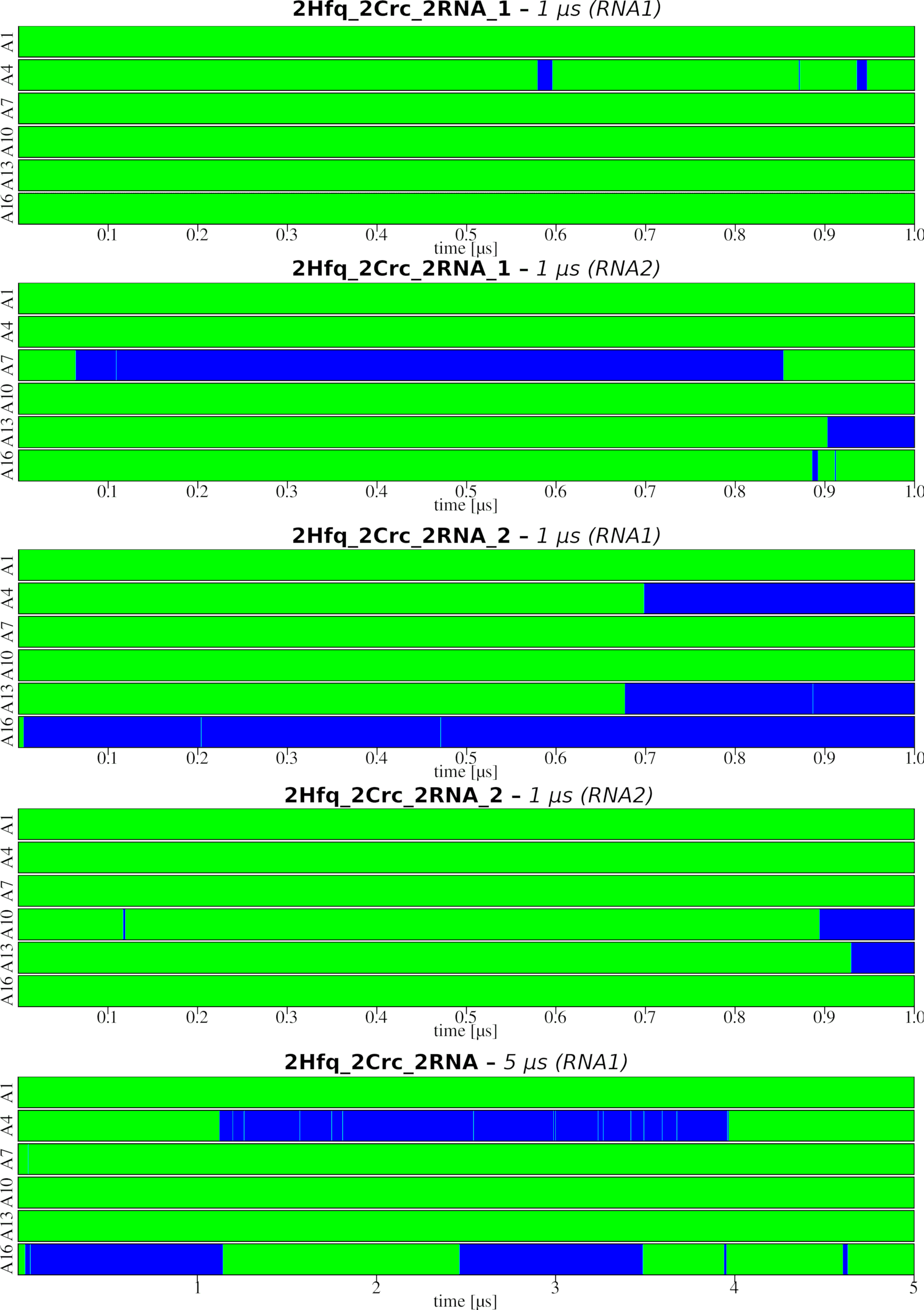


Figure S2. (Continuation from previous page)


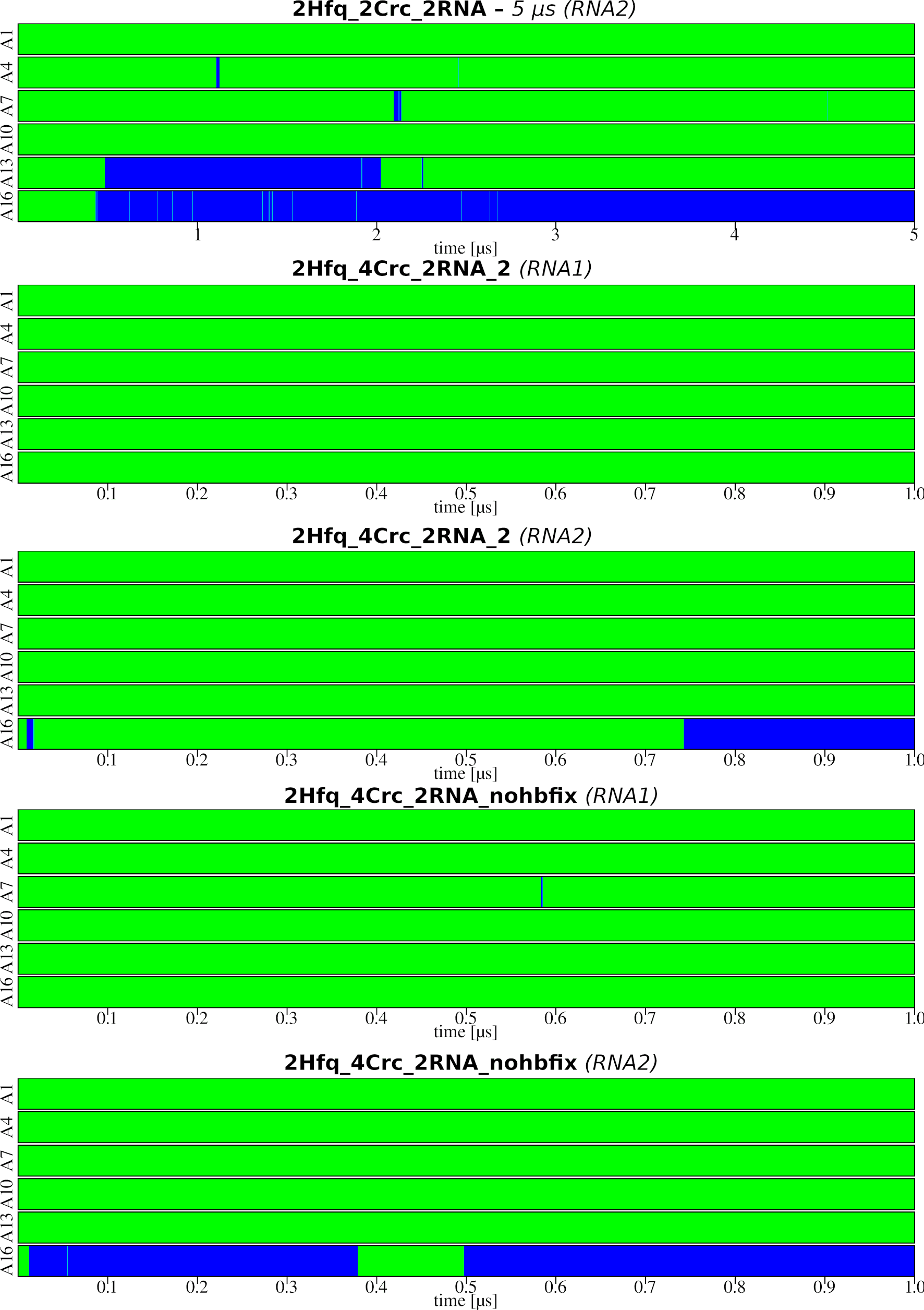


Figure S2. (Continuation from previous page)


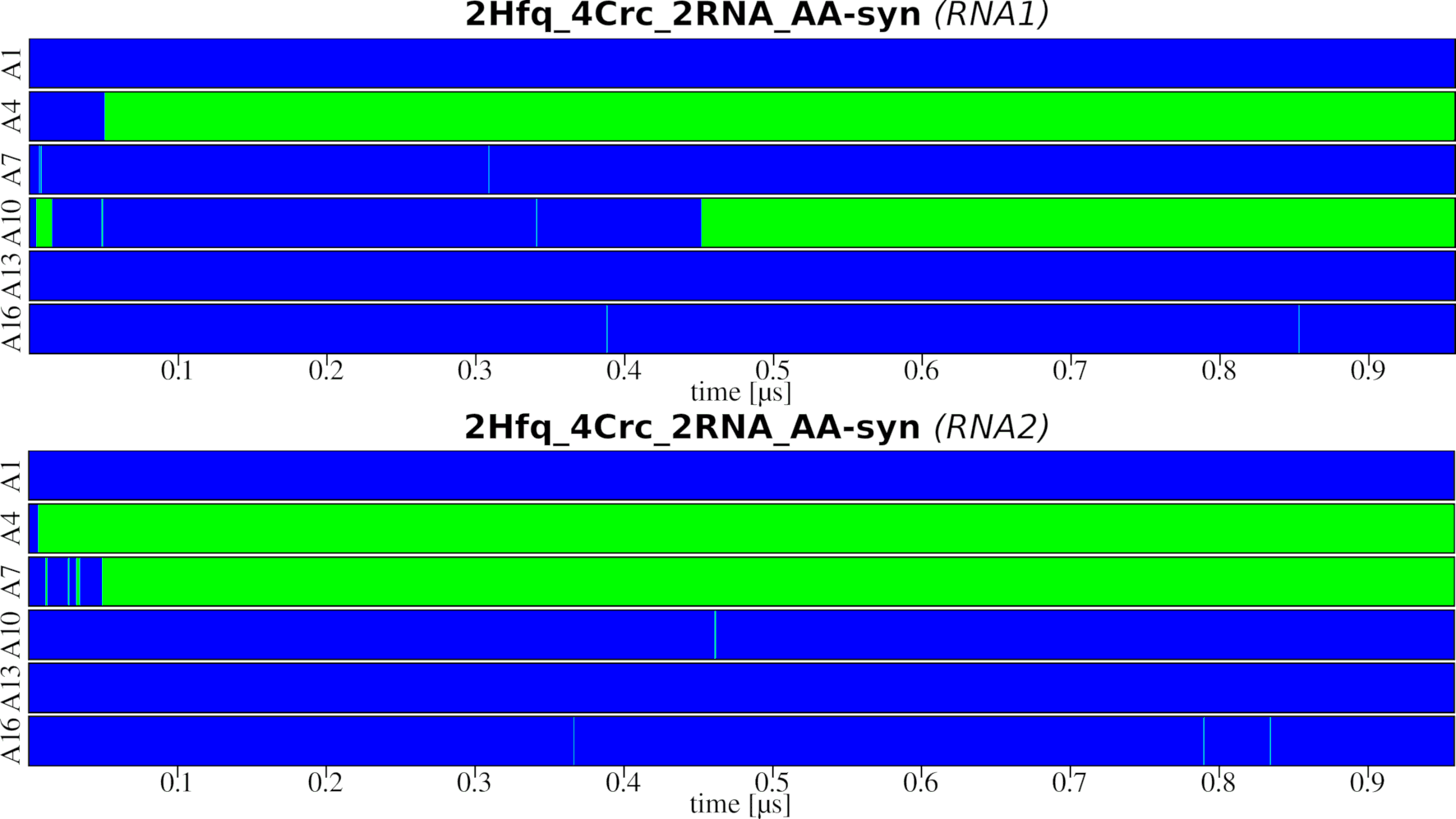


Figure S2. (Continuation from previous page)


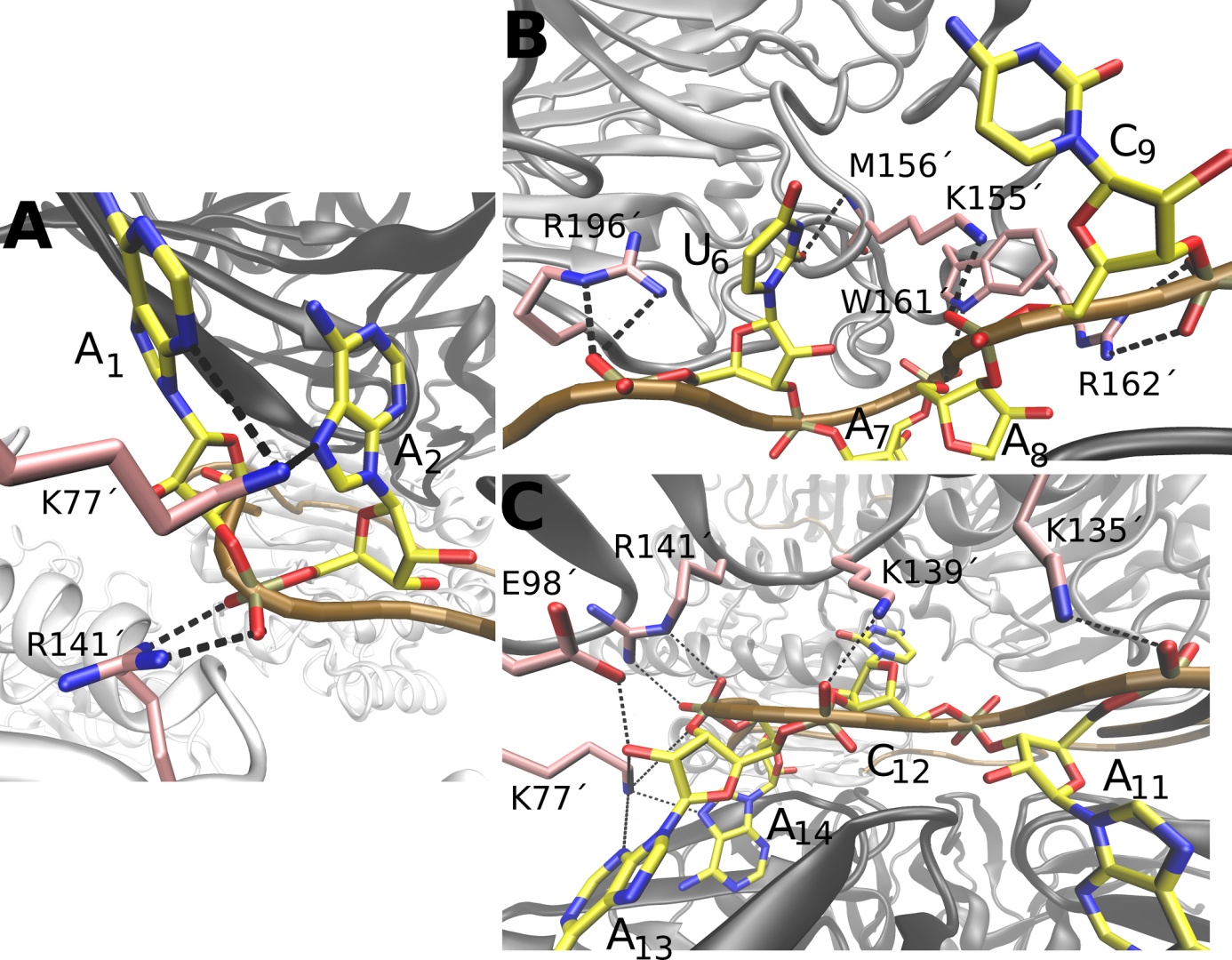


Figure S3. Native protein/RNA interactions between the Crc and RNA. The interactions in (**A**, **B**) are seen in all systems while those shown in (**C**) are specific for complexes with three and four Crc proteins. The carbon atoms are colored yellow and pink in RNA and Crc proteins, respectively. The black lines indicate H-bonds.


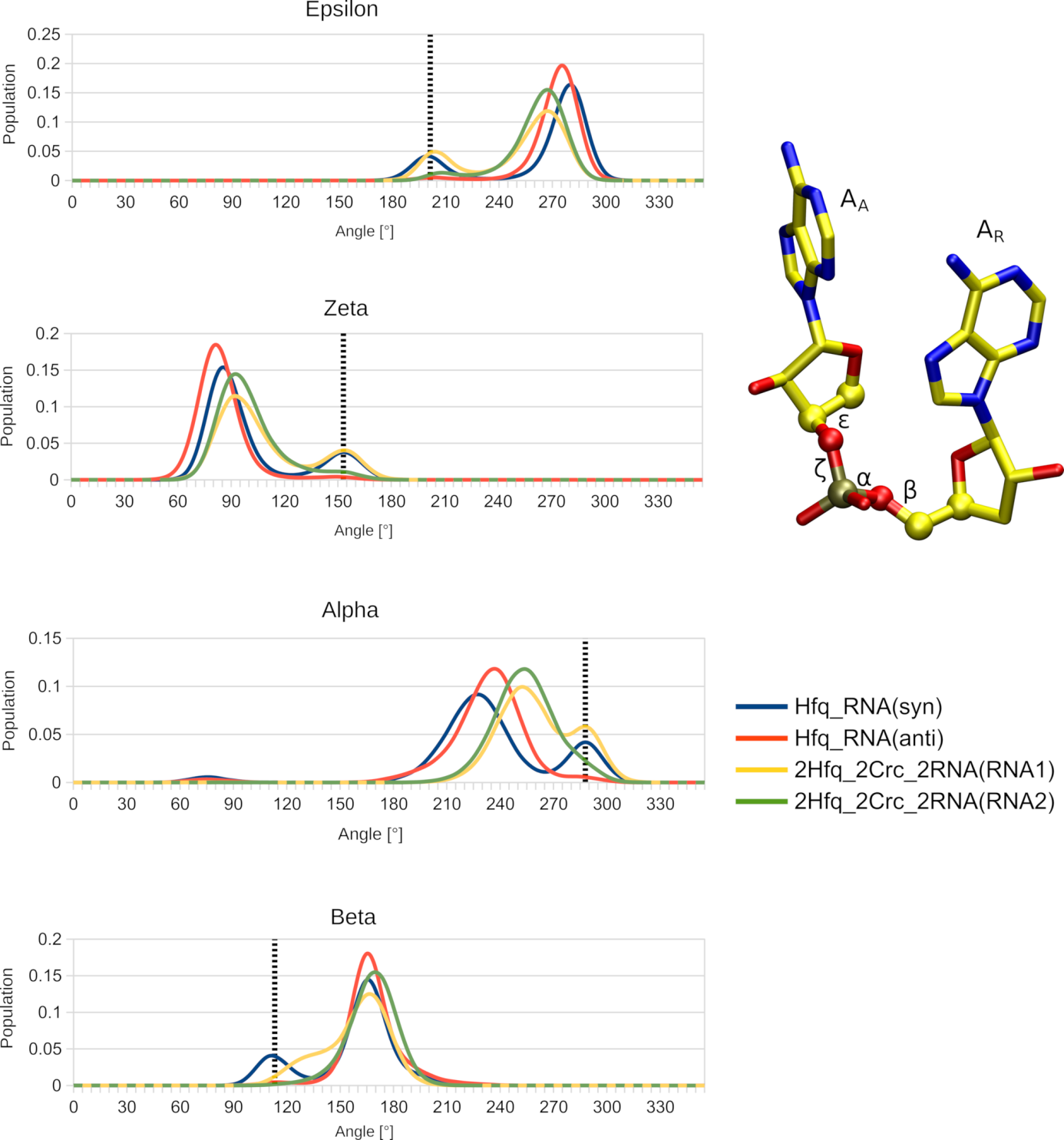


Figure S4. Histograms of populations of selected backbone torsions between A_4_ (A_A_) and A_5_ (A_R_) nucleotides in selected MD simulations. Highly similar values were observed for the other A_A_ nucleotides. Separate populations corresponding to A_4_ in *syn* and *anti*, respectively, are stated for the Hfq_RNA (10 μs) simulation. For 2Hfq_2Crc_2RNA (5 μs), the RNA1 contained mixture of *syn* and *anti* A_4_ while RNA2 had solely *anti* A_4_ (see main text Table 2 and Figure 3). There is a minor dihedral population which is accessible and characteristic only for the *syn* conformation (indicated by dashed vertical black line in each graph). This difference between *syn* and *anti* conformations of A_A_ seen in simulations is rather subtle and does not represent a full shift from one backbone suite into another ([14](#_ENREF_14)). Nevertheless, it could indirectly promote the *anti* conformation as the non-specific contacts with Crc restrict the available conformational space for such dihedral angle transitions.


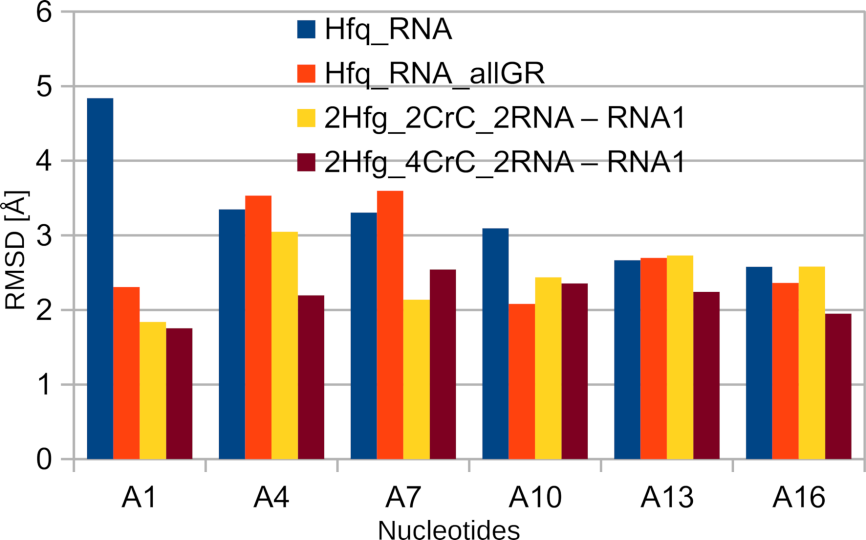


Figure S5. Sum of the total RNA backbone atom fluctuations of the individual A_A_ nucleotides in selected simulations, as indicated by the first ten eigenvectors obtained by the principal component analysis (see Methods). The calculated segment of RNA backbone included C5′, C4′, C3′ and O3′ atoms of the A_A_ nucleotides as well as the P, OP1, OP2, and O5′ atoms of the phosphate group immediately downstream of the A_A_ nucleotides.


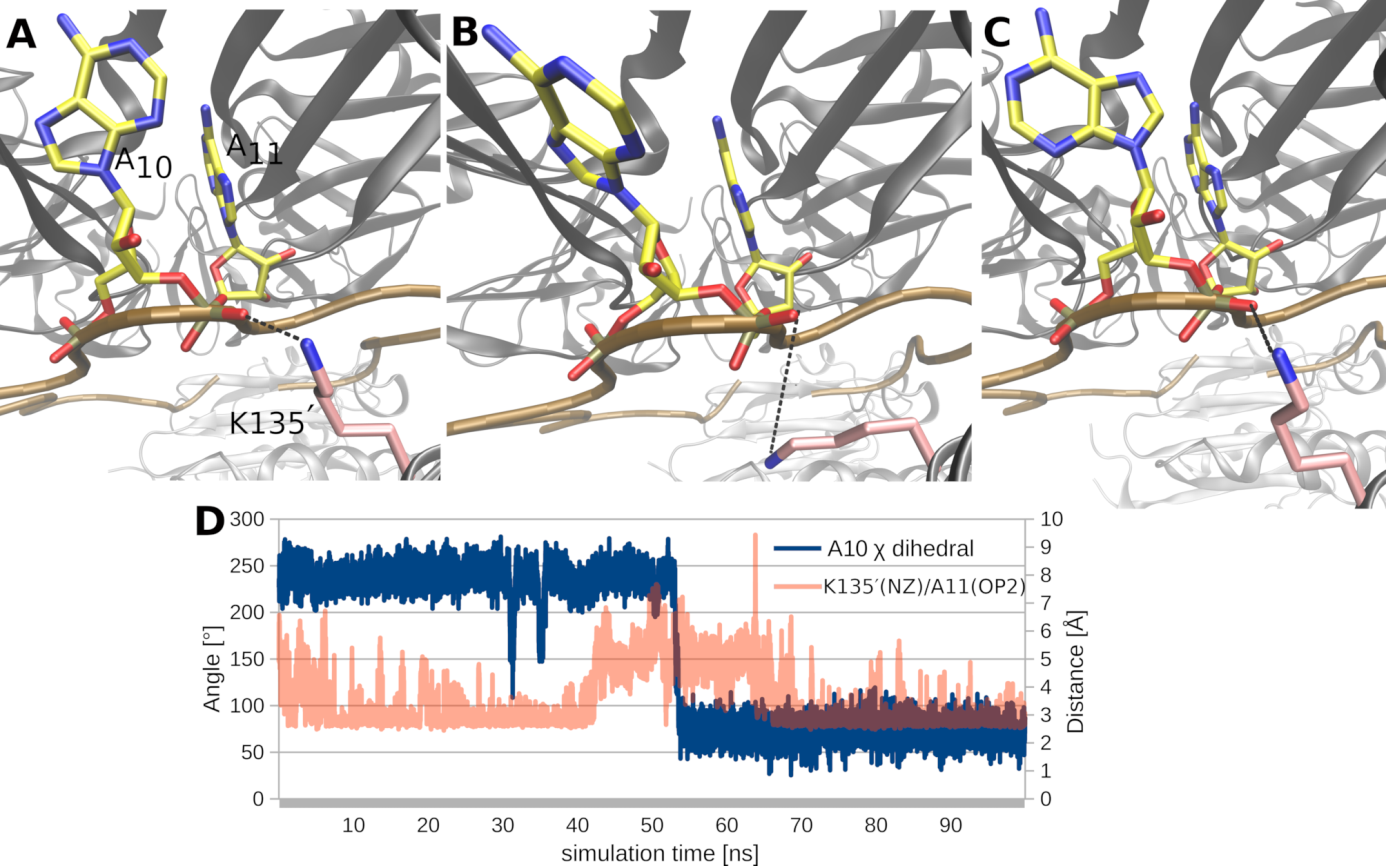


Figure S6. Example of an A_A_ *anti*🡪*syn* flip observed in 2Hfq_4Crc_2RNA_A_A_-*syn*_1 simulations facilitated by temporary loss of local Crc/RNA interaction. (**A**) Anti conformation. (**B**) Intermediate state. (**C**) Syn conformation. (D) Time development of the A_11_ χ dihedral and the K135′(NZ)/A_11_(OP2) interatomic distance. Only the portion of the simulation where the *anti*🡪*syn* flip occurred is shown.


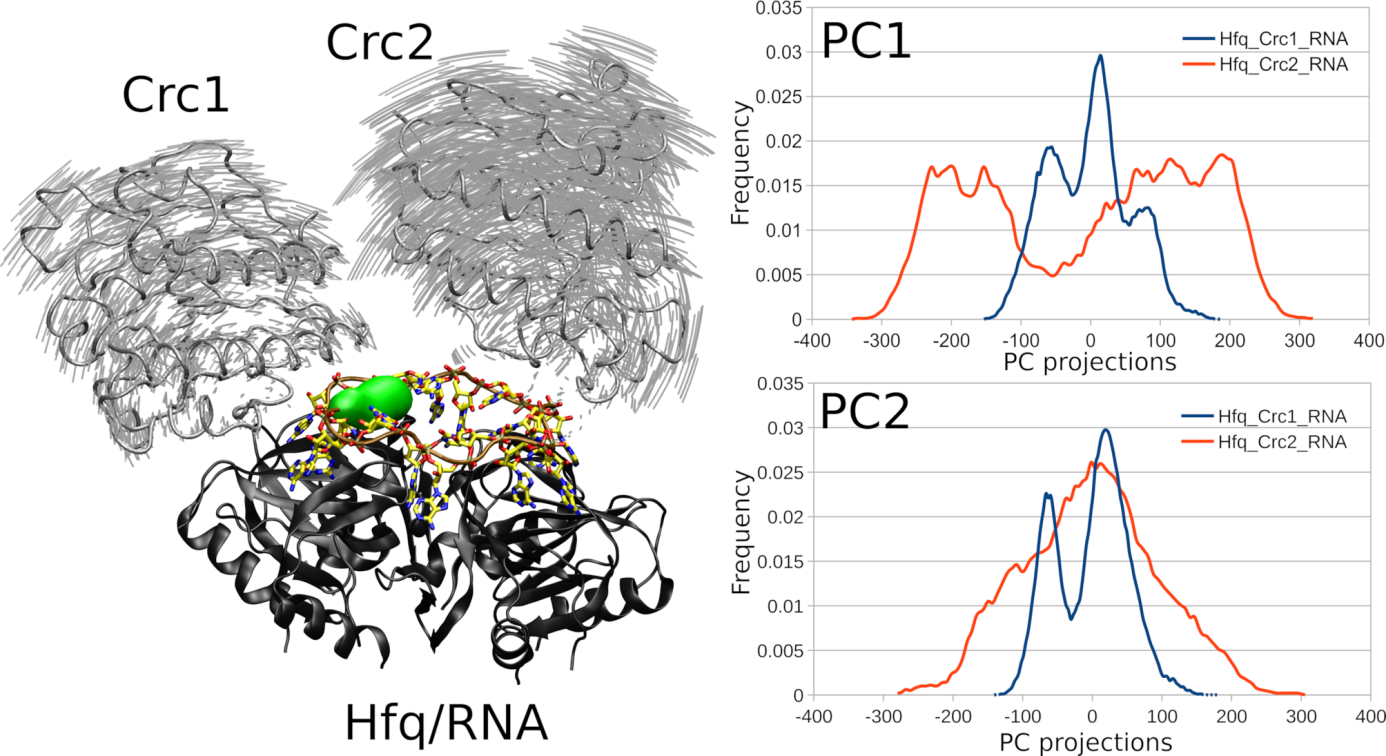


Figure S7. Global movement in the quaternary Hfq/RNA/Crc assembly as indicated by projection of the first 10 principal components of motion obtained by PCA analysis (see main text Methods). Graphs on the right show the histograms of projections of the first two principal components of motion corresponding to the inter-protein movements. In systems containing either only Crc1 or Crc2, the motions were considerably attenuated for the Crc1 protein which specifically recognizes the G_18_ nucleotide (green blob) compared to the Crc2 protein. The direction of motions and their magnitude in the individual Crc proteins is indicated by gray lines. The PCA was calculated over all heavy protein atoms.


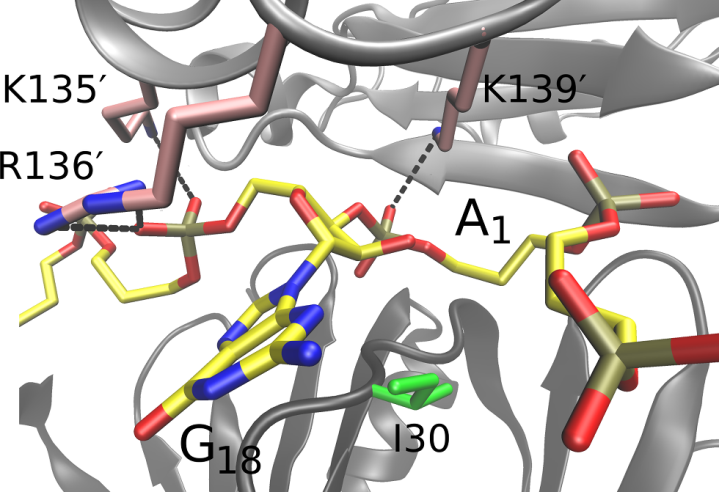


Figure S8. Circularized RNA in simulations of the quaternary complex. The circularization, i.e. covalent connection between A_1_ and G_18_, abolished the base-specific interaction between G_18_ and K139′ in favor of non-specific interaction with the newly-formed phosphate.

# Supporting Tables

Table S1. List of protein/RNA H-bonds and vdW interactions of the A_A_ nucleotides with the Hfq protein and their populations in selected simulations which were extended up to 5 or 10 μs.

| **interaction** | | **populations in simulations^a^** | | | | | |
| --- | --- | --- | --- | --- | --- | --- | --- |
| **native interactions – H-bonds** | | | | | | | |
| **simulation name** | **Hfq_RNA** | | **Hfq_RNA_allG_R_** | **2Hfg_2Crc_2RNA** | | **2Hfg_4Crc_2RNA** | |
| **K31(N)/A_4_(OP2)** | 100% | | 100% | 100% | 100% | 100% | 100% |
| **K31(N)/A_7_(OP2)** | 100% | | 100% | 98% | 99% | 100% | 98% |
| **K31(N)/A_10_(OP2)** | 100% | | 100% | 100% | 100% | 100% | 100% |
| **K31(N)/A_13_(OP2)** | 100% | | 100% | 98% | 100% | 100% | 100% |
| **K31(N)/A_16_(OP2)** | 100% | | 100% | 100% | 100% | 100% | 100% |
| **Q52(NE2)/A_1_(N1)*** | 4% | | 2% | 64% | 37% | 53% | 55% |
| **Q52(NE2)/A_4_(N1)*** | 26% | | 3% | 39% | 90% | 90% | 86% |
| **Q52(NE2)/A_7_(N1)*** | 73% | | > 1% | 91% | 90% | 91% | 85% |
| **Q52(NE2)/A_10_(N1)*** | 14% | | 20% | 86% | 91% | 84% | 90% |
| **Q52(NE2)/A_13_(N1)*** | 92% | | 2% | 97% | 65% | 59% | 66% |
| **Q52(NE2)/A_16_(N1)*** | 4% | | 12% | 9% | 6% | 44% | 15% |
| **Q33(N)/A_1_(N7)*** | 3% | | 4% | 98% | 100% | 98% | 99% |
| **Q33(N)/A_4_(N7)*** | 36% | | 9% | 40% | 96% | 93% | 97% |
| **Q33(N)/A_7_(N7)*** | 70% | | 3% | 97% | 95% | 96% | 97% |
| **Q33(N)/A_10_(N7)*** | 18% | | 51% | 97% | 97% | 96% | 96% |
| **Q33(N)/A_13_(N7)*** | 93% | | 14% | 97% | 67% | 99% | 98% |
| **Q33(N)/A_16_(N7)*** | 44% | | 18% | 55% | 8% | 78% | 81% |
| **Q33(O)/A_1_(N6)** | 100% | | 100% | 100% | 100% | 100% | 100% |
| **Q33(O)/A_4_(N6)** | 99% | | 99% | 97% | 95% | 97% | 99% |
| **Q33(O)/A_7_(N6)** | 100% | | 99% | 98% | 97% | 98% | 99% |
| **Q33(O)/A_10_(N6)** | 99% | | 99% | 97% | 98% | 98% | 99% |
| **Q33(O)/A_13_(N6)** | 98% | | 98% | 98% | 97% | 100% | 98% |
| **Q33(O)/A_16_(N6)** | 98% | | 98% | 98% | 98% | 99% | 98% |
| **non-native interactions – H-bonds** | | | | | | | |
| **Q52(OE1)/A_1_(N6)*** | 84% | | 87% | 8% | 0% | 8% | 0% |
| **Q52(OE1)/A_4_(N6)*** | 52% | | 88% | 50% | 5% | 11% | 0% |
| **Q52(OE1)/A_7_(N6)*** | 87% | | 84% | 8% | 25% | 4% | 25% |
| **Q52(OE1)/A_10_(N6)*** | 55% | | 38% | 19% | 45% | 13% | 10% |
| **Q52(OE1)/A_13_(N6)*** | 91% | | 43% | 95% | 47% | 13% | 0% |
| **Q52(OE1)/A_16_(N6)*** | 65% | | 92% | 44% | 90% | 62% | 22% |
| **Q33(N)/A_1_(N1)*** | 94% | | 94% | 0% | 0% | 0% | 0% |
| **Q33(N)/A_4_(N1)*** | 60% | | 89% | 55% | 0% | 0% | 0% |
| **Q33(N)/A_7_(N1)*** | 24% | | 94% | 0% | 1% | 0% | 0% |
| **Q33(N)/A_10_(N1)*** | 78% | | 46% | 0% | 0% | 0% | 0% |
| **Q33(N)/A_13_(N1)*** | 4% | | 81% | 0% | 30% | 0% | 0% |
| **Q33(N)/A_16_(N1)*** | 53% | | 80% | 43% | 90% | 20% | 17% |
| **Q52(NE2)/A_1_(N7)*** | 82% | | 91% | 0% | 0% | 0% | 0% |
| **Q52(NE2)/A_4_(N7)*** | 46% | | 87% | 52% | 0% | 0% | 0% |
| **Q52(NE2)/A_7_(N7)*** | 19% | | 86% | 0% | 0% | 0% | 0% |
| **Q52(NE2)/A_10_(N7)*** | 52% | | 31% | 0% | 0% | 0% | 0% |
| **Q52(NE2)/A_13_(N7)*** | 3% | | 44% | 0% | 30% | 0% | 0% |
| **Q52(NE2)/A_16_(N7)*** | 43% | | 80% | 42% | 86% | 21% | 15% |
| **native interactions – vdW interactions** | | | | | | | |
| **A_1_/L32** | 100% | | 100% | 100% | 100% | 100% | 100% |
| **A_4_/L32** | 100% | | 100% | 100% | 100% | 100% | 100% |
| **A_7_/L32** | 99% | | 100% | 100% | 100% | 100% | 100% |
| **A_10_/L32** | 100% | | 100% | 100% | 100% | 100% | 100% |
| **A_13_/L32** | 100% | | 99% | 100% | 100% | 100% | 100% |
| **A_16_/L32** | 100% | | 100% | 100% | 100% | 100% | 100% |

^a^For H-bonds, the number in the table represents percentage of all simulation frames in which the specified hydrogen bond is present. The hydrogen bond was considered present when the intermolecular distance between the heavy atoms was below 3.5 Å and its donor-hydrogen-acceptor angle was larger than 120°. For A_A_/L30 vdW interactions, the number represents percentage of all simulation ensemble frames in which the distance between the geometrical centers of the base and side chain was less than 6 Å. The reported vdW interactions occurred solely between the nucleobases and the amino acid side chains. In systems with two RNA molecules, the interactions of the individual RNA chains are listed in separate columns. The interactions marked with asterisk were only present with the base of the A_A_ nucleotide either in *syn* or *anti* conformation.

Table S2. List of protein/RNA H-bonds and stacking interactions of the A_R_ and G_R_ nucleotides with the Hfq protein and their populations in selected simulations which were extended up to 5 or 10 μs.

| **interaction** | **populations in simulations^a^** | | | | | |
| --- | --- | --- | --- | --- | --- | --- |
| **native interactions – H-bonds** | | | | | | |
| **simulation name** | **Hfq_RNA** | **Hfq_RNA_allG_R_** | **2Hfg_2Crc_2RNA** | | **2Hfg_4Crc_2RNA** | |
| **G29(O)/A_2_(O2′)** | 51% | 57% | 43% | 67% | 99% | 57% |
| **G29(O)/A_5_(O2′)** | 42% | 26% | 97% | 97% | 97% | 97% |
| **G29(O)/A_8_(O2′)** | 46% | 50% | 100% | 98% | 99% | 98% |
| **G29(O)/A_11_(O2′)** | 63% | 51% | 45% | 46% | 82% | 97% |
| **G29(O)/A_14_(O2′)** | 26% | 45% | 29% | 24% | 42% | 57% |
| **G29(O)/G_17_(O2′)** | 6% | 4% | 18% | 6% | 2% | 7% |
| **T61(OG1)/A_2_(N1)** | 75% | 98% | 94% | 65% | 91% | 74% |
| **T61(OG1)/A_5_(N1)** | 71% | 96% | 96% | 94% | 94% | 90% |
| **T61(OG1)/A_8_(N1)** | 23% | 90% | 95% | 89% | 95% | 83% |
| **T61(OG1)/A_11_(N1)** | 77% | 89% | 85% | 57% | 91% | 96% |
| **T61(OG1)/A_14_(N1)** | 27% | 90% | 8% | 53% | 61% | 78% |
| **T61(OG1)/G_17_(N1)** | 89% | 93% | 63% | 89% | 95% | 98% |
| **Q52(OE1)/A_2_(N6)** | 8% | 48% | 13% | 97% | 44% | 96% |
| **Q52(OE1)/A_5_(N6)** | 38% | 45% | 15% | 71% | 54% | 83% |
| **Q52(OE1)/A_8_(N6)** | 7% | 42% | 31% | 59% | 23% | 45% |
| **Q52(OE1)/A_11_(N6)** | 32% | 50% | 34% | 45% | 45% | 38% |
| **Q52(OE1)/A_14_(N6)** | 3% | 55% | 0% | 12% | 77% | 97% |
| **Q52(NE)/G_17_(O6)** | 38% | 25% | 71% | 37% | 55% | 82% |
| **native interactions – stacking** | | | | | | |
| **A_2_/Y25** | 90% | 100% | 100% | 100% | 100% | 100% |
| **A_5_/Y25** | 99% | 100% | 100% | 100% | 100% | 100% |
| **A_8_/Y25** | 99% | 100% | 100% | 100% | 100% | 100% |
| **A_11_/Y25** | 98% | 100% | 100% | 96% | 100% | 100% |
| **A_14_/Y25** | 95% | 100% | 97% | 100% | 100% | 100% |
| **G_17_/Y25** | 100% | 100% | 100% | 100% | 100% | 100% |

^a^See the Footnote ^a^ of Table S1. For A_A_/Y25 stacking interactions, the number represents percentage of all simulation ensemble frames in which the distance between the geometrical centers of the base and the side chain was less than 5 Å.

Table S3. Transitions between the *anti* and *syn* conformations of individual A_A_ nucleotides in selected MD simulations not included in the main text Table 2.^a^

| **simulation name**  **and length** | | | **I: number of transitions / syn population^b^**  **II: time until first transition (ns)^c^**  **III: average lifetimes of syn / anti states (ns)^d^** | | | | | |
| --- | --- | --- | --- | --- | --- | --- | --- | --- |
|  | | **A_1_** | | **A_4_** | **A_7_** | **A_10_** | **A_13_** | **A_16_** |
| **Hfq_RNA_1 – *1 μs*** | **I** | 1 / 0.23 | | 7 / 0.35 | 5 / 0.16 | 10 / 0.18 | 1 / 0.60 | 1 / 0.63 |
|  | **II** | 774 | | 165 | 818 | 160 | 402 | 373 |
|  | **III** | – | | 71 / 130 | 55 / 279 | 36 / 136 | – | – |
| **Hfq_RNA_2 – *1 μs*** | **I** | 1 / 0.96 | | 0 / 0.00 | 4 / 0.64 | 2 / 0.57 | 3 / 0.67 | 4 / 0.62 |
|  | **II** | 35 | | – | 313 | 139 | 238 | 36 |
|  | **III** | – | | – | 321 / 119 | 574 / 213 | 337 / 163 | 308 / 128 |
| **Hfq_RNA_allG_R__1 – *2 μs*** | **I** | 1 / 0.91 | | 7 / 0.41 | 0 / 0.00 | 4 / 0.13 | 2 / 0.77 | 1 / 0.90 |
|  | **II** | 174 | | 453 | – | 611 | 459 | 198 |
|  | **III** | – | | 203 / 238 | – | 132 / 579 | 1543 / 229 | – |
| **Hfq_RNA_allG_R__2 – *2 μs*** | **I** | 1 / 0.83 | | 1 / 0.81 | 5 / 0.66 | 5 / 0.63 | 7 / 0.13 | 5 / 0.85 |
|  | **II** | 338 | | 376 | 363 | 87 | 900 | 34 |
|  | **III** | – | | – | 440 / 227 | 422 / 245 | 52 / 349 | 570 / 97 |
| **Hfq_RNA_nohbfix_1 – *2 μs*** | **I** | 8 / 0.54 | | 15 / 0.73 | 3 / 0.15 | 6 / 0.70 | 13 / 0.77 | 1 / 0.81 |
|  | **II** | 702 | | 39 | 1518 | 141 | 145 | 375 |
|  | **III** | 136 / 153 | | 82 / 30 | 77 / 423 | 233 / 86 | 118 / 39 | – |
| **Hfq_RNA_nohbfix_2 – *2 μs*** | **I** | 3 / 0.79 | | 6 / 0.96 | 2 / 0.01 | 9 / 0.74 | 14 / 0.61 | 1 / 0.59 |
|  | **II** | 1470 | | 606 | 477 | 224 | 138 | 1171 |
|  | **III** | 262 / 108 | | 275 / 13 | 19 / 991 | 213 / 73 | 153 / 110 | – |
| **Hfq_RNA_2kcal-mol_hbfix**  **– *2 μs*** | **I** | 5 / 0.92 | | 8 / 0.43 | 0 / 0.0 | 5 / 0.82 | 3 / 0.31 | 2 / 0.06 |
|  | **II** | 107 | | 910 | – | 267 | 1276 | 1373 |
|  | **III** | 616 / 51 | | 145 / 162 | – | 550 / 88 | 309 / 691 | 114 / 943 |
| **2Hfq_2Crc_2RNA_1 – *1 μs*** | **I** | 0 / 0.00  0 / 0.00 | | 4 / 0.03  0 / 0.00 | 0 / 0.00  2 / 0.79 | 0 / 0.00  0 / 0.00 | 0 / 0.00  1 / 0.10 | 0 / 0.00  4 / 0.01 |
|  | **II** | –  – | | 580  – | –  66 | –  – | –  903 | –  887 |
|  | **III** | –  – | | 11 / 242  – | –  791 / 105 | –  – | –  – | –  5 / 331 |
| **2Hfq_2Crc_2RNA_2 – *1 μs*** | **I** | 0 / 0.00  0 / 0.00 | | 1 / 0.30  0 / 0.00 | 0 / 0.00  0 / 0.00 | 0 / 0.00  3 / 0.11 | 1 / 0.07  1 / 0.32 | 1 / 0.99  0 / 0.00 |
|  | **II** | –  – | | 701  – | –  – | –  118 | 676  930 | 6  – |
|  | **III** | –  – | | –  – | –  – | –  55 / 446 | –  – | –  – |
| **2Hfq_4Crc_2RNA_2 – *1 μs*** | **I** | 0 / 0.00  0 / 0.00 | | 0 / 0.00  0 / 0.00 | 0 / 0.00  0 / 0.00 | 0 / 0.00  0 / 0.00 | 0 / 0.00  0 / 0.00 | 0 / 0.00  3 / 0.27 |
|  | **II** | –  – | | –  – | –  – | –  – | –  – | –  9 |
|  | **III** | –  – | | –  – | –  – | –  – | –  – | –  133 / 367 |
| **2Hfq_4Crc_2RNA_noHBfix – *1 μs*** | **I** | 0 / 0.00  0 / 0.00 | | 0 / 0.00  0 / 0.00 | 0 / 0.00  0 / 0.00 | 0 / 0.00  0 / 0.00 | 0 / 0.00  0 / 0.00 | 0 / 0.00  3 / 0.87 |
|  | **II** | –  – | | –  – | –  – | –  – | –  – | –  15 |
|  | **III** | –  – | | –  – | –  – | –  – | –  – | –  435 / 65 |
| **2Hfq_4Crc_2RNA_A_A_-*syn* – *1 μs*** | **I** | 0 / 1.00  0 / 1.00 | | 1 / 0.07  1 / 0.06 | 0 / 1.00  3 / 0.05 | 3 / 0.44  0 / 0.00 | 0 / 1.00  0 / 1.00 | 0 / 1.00  0 / 1.00 |
|  | **II** | –  – | | 57  65 | –  34 | 5  – | –  – | –  – |
|  | **III** | –  – | | –  – | –  50 / 455 | 220 / 174  – | –  – | –  – |

^a^The two lines in the “2RNA” simulations each describe one of the two RNA molecules contained in these systems. Crc’s sterically obstruct flips of A_1_ in 2Crc and of A_1_ and A_13_ in 4Crc systems, respectively. ^b^Number of *syn*/*anti* transitions (in any direction) and the populations of the two states with one and zero corresponding to all-*syn* and all-*anti*, respectively. The A_A_ nucleotide was considered to be in *syn* and *anti* when its χ dihedral angle was -30°–150° and 150°–330°, respectively. We disregarded transitions lasting less than 300 ps. ^c^Simulation time in which the first *syn*/*anti* transition (in either direction) occurred. The “–” symbol indicates that no transition occurred. ^d^Average simulation time that A_A_ nucleotide remained in *syn* and *anti*, respectively, before flipping. The lifetimes are not stated when there was only a single or no transition observed.

Table S4. List of H-bonds and stacking interactions between RNA and Crc and their relative stability in selected simulations which were extended up to 5 μs and are presented in the main text.

| **H-bond** | **relative stability in simulations^a^** | | | | | | | | |
| --- | --- | --- | --- | --- | --- | --- | --- | --- | --- |
| **Interactions present in all quaternary complexes** | | | | | | | | | |
|  | **2Hfq/2Crc/2RNA** | | **2Hfq/4Crc/2RNA** | | | **2Hfq/4Crc/2RNA_ A_A_-*syn*_1** | | **2Hfq/4Crc/2RNA_ A_A_-*syn*_2** | |
| **H-bonds** | | | | | | | | | |
| D98**´**(OD)/A_1_(O2’) | 93% | 100% | 100% | | 100% | 96% | 100% | 97% | 100% |
| K77**´**(NZ)/A_1_(N3) | 1% | 19% | 3% | | 28% | 0% | 0% | 0% | 0% |
| K77**´**(NZ)/A_2_(N7) | 4% | 40% | 33% | | 82% | 5% | 29% | 2% | 42% |
| M156**´**(N)/U_6_(O2) | 95% | 93% | 97% | | 82% | 33% | 94% | 97% | 92% |
| W161**´**(NE1)/A_8_(O5’) | 94% | 76% | 91% | | 84% | 38% | 84% | 53% | 94% |
| R141**´**(sc)/A_2_(OP) | 72% | 64% | 75% | | 96% | 96% | 99% | 88% | 97% |
| K139**´**(NZ)/A_2_(OP) | 99% | 35% | 59% | | 7% | 6% | 11% | 84% | 24% |
| R140**´**(N)/A_3_(OP) | 42% | 0% | 74% | | 0% | 0% | 6% | 0% | 0% |
| R196**´**(sc)/U_6_(OP) | 85% | 77% | 90% | | 76% | 6% | 11% | 76% | 63% |
| K155**´**(NZ)/C_9_(OP) | 70% | 67% | 66% | | 68% | 63% | 32% | 64% | 55% |
| R162**´**(*sc*)/A_10_(OP) | 100% | 85% | 100% | | 86% | 45% | 99% | 100% | 93% |
| H131**´**(NE2)/G_17_(OP) | 5% | 69% | 35% | | 16% | 82% | 84% | 5% | 18% |
| **Stacking** | | | | | | | | | |
| A_3_/R140**´** | 19% | 42% | 100% | | 28% | 99% | 99% | 91% | 98% |
| A_3_/R196**´** | 23% | 39% | 8% | | 47% | 49% | 100% | 21% | 12% |
| **Interactions present only in quaternary complexes with three or four Crc’s** | | | | | | | | | |
|  | **2Hfq/4Crc/2RNA** | | | | | **2Hfq/4Crc/2RNA_ A_A_-*syn*_1** | | **2Hfq/4Crc/2RNA_ A_A_-*syn*_2** | |
| **H-bonds** | | | | | | | | | |
| R138**´**(sc)/C_9_(O2) | 8% | | | 20% | | 3% | 8% | 2% | 11% |
| R138**´**(sc)/C_9_(N3) | 11% | | | 26% | | 5% | 8% | 2% | 16% |
| R140**´**(N)/C_12_(O2) | 26% | | | 92% | | 2% | 16% | 70% | 20% |
| E98**´**(OD)/A_13_(O2’) | 100% | | | 100% | | 99% | 67% | 98% | 100% |
| K77**´**(NZ)/A_13_(N3) | 26% | | | 30% | | 0% | 0% | 0% | 0% |
| K77**´**(NZ)/A_14_(N7) | 60% | | | 85% | | 4% | 1% | 3% | 7% |
| K135**´**(NZ)/A_11_(OP) | 73% | | | 88% | | 74% | 65% | 67% | 45% |
| R138**´**(sc)/C_12_(OP) | 38% | | | 50% | | 65% | 13% | 62% | 39% |
| K139**´**(NZ)/A_13_(OP) | 71% | | | 72% | | 98% | 62% | 23% | 91% |
| R141**´**(sc)/A_14_(OP) | 48% | | | 100% | | 70% | 34% | 52% | 75% |
| R140**´**(sc)/G_15_(OP) | 35% | | | 52% | | 30% | 23% | 26% | 19% |
| **Stacking** | | | | | | | | | |
| C_9_/R138**´** | 76% | | | 60% | | 25% | 34% | 93% | 81% |
| G_15_/R140**´** | 48% | | | 16% | | 25% | 13% | 55% | 60% |

^a^See the Footnote ^a^ of Table S1. For base stacking interactions with the arginine side-chains, the number represents percentage of all simulation ensemble frames in which the distance between the geometrical centers of the base and of the arginine guanidinium group was less than 5 Å while the arginine side chain was not forming H-bond with the base.

Table S5. List of protein/RNA H-bonds, salt-bridges and vdW interactions of the N nucleotides (except G_18_) with the Hfq protein and their relative stability in selected simulations that were extended up to 5 or 10 μs.

| **Interaction** | **relative stability in simulations^a^** | | | | | |
| --- | --- | --- | --- | --- | --- | --- |
| **H-bond or salt-bridge** | | | | | | |
| **simulation name** | **Hfq_RNA** | **Hfq_RNA_allG_R_** | **2Hfg_2Crc_2RNA** | | **2Hfg_4Crc_2RNA** | |
| **N28(O)/A_3_(N6)** | 79% | 89% | 36% | 0% | 0% | 62% |
| **N28(O)-U_6_(N6)** | 50% | 55% | 0% | 0% | 0% | 0% |
| **N28(O)/C_9_(N6)** | 92% | 85% | 0% | 0% | 0% | 0% |
| **N28(O)/C_12_(N4)** | 78% | 85% | 37% | 40% | 29% | 0% |
| **N28(O)-G_15_(O6)** | 43% | 67% | 4% | 12% | 28% | 52% |
| **vdW interaction** | | | | | | |
| **A_3_/I30** | 97% | 98% | 87% | 0% | 0% | 71% |
| **U_6_/I30** | 89% | 95% | 1% | 1% | 4% | 3% |
| **C_9_/I30** | 99% | 96% | 18% | 49% | 3% | 4% |
| **C_12_/I30** | 93% | 97% | 91% | 99% | 59% | 2% |
| **G_15_/I30** | 97% | 98% | 77% | 97% | 83% | 96% |

^a^See the Footnote ^a^ of Table S1. For base vdW interaction with I30 side-chain, the number represents percentage of all simulation ensemble frames in which the distance between the geometrical centers of the base and the side chain was less than 6 Å. The binding of guanine and uracil in this pocket was generally less stable than of the adenine and cytosine, since it could be disrupted during exchanges of the bound ion which stabilizes the interaction with N28(O) (main text Figure 6). However, the vdW interaction with I30 was not lost even during these ion exchanges.

Table S6. List of selected protein/RNA H-bonds and vdW interactions of the RNA nucleotides with the Hfq protein and their populations in the control Hfq_RNA_I30A simulations.

| **Interaction** | **relative stability in simulations^a^** |
| --- | --- |
| **Non-native interactions – H-bonds** | |
| **N28(O)/A_3_(N6)** | 12% |
| **N28(O)-U_6_(N6)** | 0% |
| **N28(O)/C_9_(N6)** | 37% |
| **N28(O)/C_12_(N4)** | 41% |
| **N28(O)-G_15_(O6)** | 22% |
| **Non-native interactions – vdW interaction** | |
| **A_3_/A30** | 13% |
| **U_6_/A30** | 0% |
| **C_9_/A30** | 52% |
| **C_12_/A30** | 42% |
| **G_15_/A30** | 42% |

^a^See the Footnote ^a^ of Table S5.

Table S7. List of H-bond and vdW interactions formed by the G_18_ nucleotide and their relative stability in selected simulations which were extended up to 5 or 10 μs.

| **Interaction** | **relative stability in simulations^a^** | | | | | | |
| --- | --- | --- | --- | --- | --- | --- | --- |
| **H-bond** | | | | | | | |
| **simulation name** | **Hfq_RNA** | **Hfq_RNA_allG_R_** | **Hfq_RNA_Crc1** | **2Hfg_2Crc_2RNA** | | **2Hfg_4Crc_2RNA** | |
| **K139´(NZ)/G_18_(O6)** | - | - | 12% | 5% | 33% | 27% | 5% |
| **K139´(NZ)/G_18_(N7)** | - | - | 13% | 1% | 26% | 8% | 4% |
| **R138´(*sc*)/G_18_(OP)** | - | - | 31% | 52% | 14% | 10% | 5% |
| **K135´(NZ)/G_18_(OP)** | - | - | 23% | 61% | 26% | 32% | 54% |
| **G_18_(N2)/A_3_(OP)** | 2% | 3% | 88% | 52% | 88% | 89% | 24% |
| **G_18_(N1)/A_3_(OP)** | 1% | 2% | 58% | 47% | 85% | 97% | 18% |
| **vdW interaction** | | | | | | | |
| **G_18_/I30** | 6% | 10% | 100% | 100% | 98% | 100% | 25% |

^a^See the Footnote ^a^ of Table S1. For vdW interaction G_18_/I30, the number represents percentage of all simulation ensemble frames in which the distance between the geometrical centers of the base and the side chain was less than 6 Å.

Table S8. List of protein/RNA H-bonds, stacking and vdW interactions of the RNA nucleotides with the Hfq protein and their relative stability in the Hfq_RNA_pol-A simulation.

| **Interaction** | **relative stability in simulations^a^** |
| --- | --- |
| **Native interactions – H-bonds** | |
| **K31(N)/A_A_(OP2)** | 99% |
| **Q52(NE2)/A_A_(N1)*** | 18% |
| **Q33(N)/A_A_(N7)*** | 41% |
| **Q33(O)/A_A_(N6)** | 93% |
| **G29(O)/A_R_(O2’)** | 52% |
| **T61(OG1)/A_R_(N1)** | 30% |
| **Q52(OE1)/A_R_(N6)** | 28% |
| **Non-native interactions – H-bonds** | |
| **Q52(OE1)/A_A_(N6)*** | 40% |
| **Q33(N)/A_A_(N1)*** | 52% |
| **Q52(NE2)/A_A_(N7)*** | 31% |
| **N28(O)/A_N_(N6)** | 72% |
| **Native interactions – stacking or vdW interaction** | |
| **A_A_/L32** | 99% |
| **A_G_/Y25** | 97% |
| **Non-native interactions – vdW interaction** | |
| **A_N_/I30** | 94% |

^a^See the Footnote ^a^ of Table S1, S2, and S4.

# Supporting References

1. Kuhrova, P., Best, R., Bottaro, S., Bussi, G., Sponer, J., Otyepka, M., and Banas, P. (2016) Computer Folding of RNA Tetraloops: Identification of Key Force Field Deficiencies. *J. Chem. Theory Comput.* **12**, 4534–4548

2. Šponer, J., Bussi, G., Krepl, M., Banáš, P., Bottaro, S., Cunha, R. A., Gil-Ley, A., Pinamonti, G., Poblete, S., Jurečka, P., Walter, N. G., and Otyepka, M. (2018) RNA Structural Dynamics as Captured by Molecular Simulations: A Comprehensive Overview. *Chem. Rev.* **118**, 4177–4338

3. Šponer, J., Krepl, M., Banáš, P., Kührová, P., Zgarbová, M., Jurečka, P., Havrila, M., and Otyepka, M. (2017) How to Understand Atomistic Molecular Dynamics Simulations of RNA and Protein–RNA Complexes? *Wiley Interdiscip. Rev.: RNA* **8**, e1405

4. Kuhrova, P., Mlynsky, V., Zgarbova, M., Krepl, M., Bussi, G., Best, R. B., Otyepka, M., Sponer, J., and Banas, P. (2019) Improving the Performance of the RNA Amber Force Field by Tuning the Hydrogen-Bonding Interactions. *J. Chem. Theory Comput.* **15**, 3288-3305

5. Bergonzo, C., Henriksen, N. M., Roe, D. R., Swails, J. M., Roitberg, A. E., and Cheatham, T. E. (2014) Multidimensional Replica Exchange Molecular Dynamics Yields a Converged Ensemble of an RNA Tetranucleotide. *J. Chem. Theory Comput.* **10**, 492-499

6. Bergonzo, C., Henriksen, N. M., Roe, D. R., and Cheatham, T. E. (2015) Highly Sampled Tetranucleotide and Tetraloop Motifs Enable Evaluation of Common RNA Force Fields. *RNA* **21**, 1578-1590

7. Szabla, R., Havrila, M., Kruse, H., and Šponer, J. (2016) Comparative Assessment of Different RNA Tetranucleotides from the DFT-D3 and Force Field Perspective. *J. Phys. Chem. B* **120**, 10635-10648

8. Bottaro, S., Bussi, G., Kennedy, S. D., Turner, D. H., and Lindorff-Larsen, K. (2018) Conformational Ensembles of RNA Oligonucleotides from Integrating NMR and Molecular Simulations. *Sci. Adv.* **4**, eaar8521

9. Mlýnský, V., Kührová, P., Kühr, T., Otyepka, M., Bussi, G., Banáš, P., and Šponer, J. (2020) Fine-Tuning of the AMBER RNA Force Field with a New Term Adjusting Interactions of Terminal Nucleotides. *J. Chem. Theory Comput.* **16**, 3936-3946

10. Condon, D. E., Kennedy, S. D., Mort, B. C., Kierzek, R., Yildirim, I., and Turner, D. H. (2015) Stacking in RNA: NMR of Four Tetramers Benchmark Molecular Dynamics. *J. Chem. Theory Comput.* **11**, 2729-2742

11. Zhao, J., Kennedy, S. D., Berger, K. D., and Turner, D. H. (2020) Nuclear Magnetic Resonance of Single-Stranded RNAs and DNAs of CAAU and UCAAUC as Benchmarks for Molecular Dynamics Simulations. *J. Chem. Theory Comput.* **16**, 1968-1984

12. Banáš, P., Mládek, A., Otyepka, M., Zgarbová, M., Jurečka, P., Svozil, D., Lankaš, F., and Šponer, J. (2012) Can We Accurately Describe the Structure of Adenine Tracts in B-DNA? Reference Quantum-Chemical Computations Reveal Overstabilization of Stacking by Molecular Mechanics. *J. Chem. Theory Comput.* **8**, 2448-2460

13. Sonnleitner, E., Wulf, A., Campagne, S., Pei, X.-Y., Wolfinger, M. T., Forlani, G., Prindl, K., Abdou, L., Resch, A., Allain, F. H.-T., Luisi, B. F., Urlaub, H., and Bläsi, U. (2018) Interplay between the Catabolite Repression Control Protein Crc, Hfq and RNA in Hfq-dependent Translational Regulation in Pseudomonas aeruginosa. *Nucleic Acids Res.* **46**, 1470-1485

14. Richardson, J. S., Schneider, B., Murray, L. W., Kapral, G. J., Immormino, R. M., Headd, J. J., Richardson, D. C., Ham, D., Hershkovits, E., Williams, L. D., Keating, K. S., Pyle, A. M., Micallef, D., Westbrook, J., and Berman, H. M. (2008) RNA backbone: Consensus All-angle Conformers and Modular String Nomenclature (An RNA Ontology Consortium Contribution). *RNA* **14**, 465-481
